# Microfluidic Crystallization of Surfactant-Free Doped Zinc Sulfide Nanoparticles for Optical Bioimaging Applications

**DOI:** 10.1021/acsami.0c13150

**Published:** 2020-09-02

**Authors:** Francesca Tajoli, Nicola Dengo, Maddalena Mognato, Paolo Dolcet, Giacomo Lucchini, Andrea Faresin, Jan-Dierk Grunwaldt, Xiaohui Huang, Denis Badocco, Michele Maggini, Christian Kübel, Adolfo Speghini, Tommaso Carofiglio, Silvia Gross

**Affiliations:** † Dipartimento di Scienze Chimiche, Università degli Studi di Padova, Via Marzolo 1, 35131 Padova, Italy; ‡ INSTM, UdR di Padova, Via Marzolo 1, 35131 Padova, Italy; § Dipartimento di Biologia, Università degli Studi di Padova, Via Bassi 58B, 35131 Padova, Italy; ∥ Karlsruher Institut für Technologie (KIT), Institut für Technische Chemie und Polymerchemie (ITCP), Engesserstr. 20, 76131 Karlsruhe, Germany; ⊥ NRG, Dipartimento di Biotecnologie, Università di Verona and INSTM, RU Verona, Strada Le Grazie 15, 37314 Verona, Italy; # Karlsruher Institut für Technologie (KIT), Institut für Nanotechnologie (INT) & Karlsruhe Nano Micro Facility (KNMF), Hermann-von-Helmholtz-Platz 1, 76344 Eggenstein-Leopoldshafen, Germany; ∇ Department of Materials and Earth Sciences, Technical University Darmstadt, Alarich-Weiss-Str. 2, 64287 Darmstadt, Germany

**Keywords:** ZnS, zinc sulfide, microfluidics, optical bioimaging, doping, luminescence, NIR emission

## Abstract

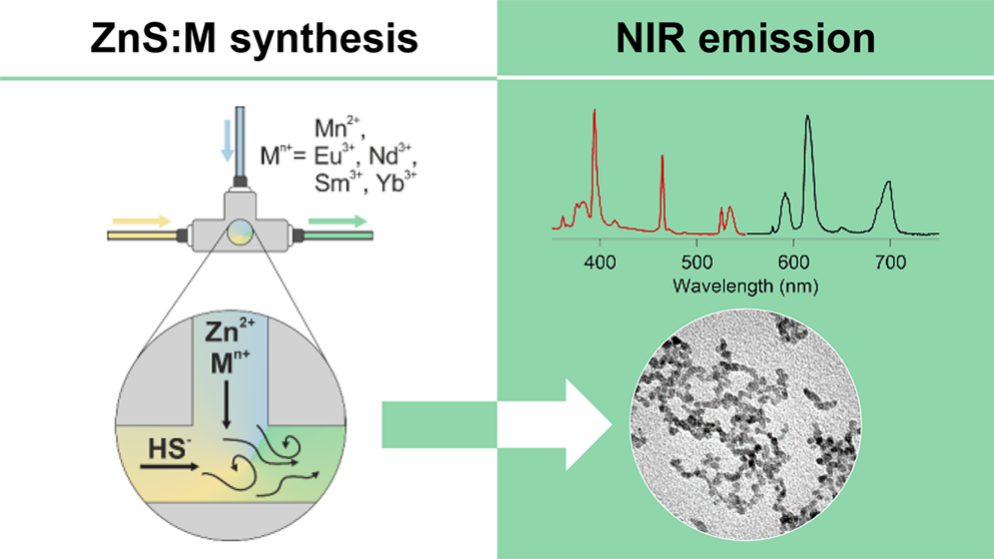

The room-temperature controlled crystallization of monodispersed ZnS nanoparticles (average size of 5 nm) doped with luminescent ions (such as Mn^2+^, Eu^3+^, Sm^3+^, Nd^3+^, and Yb^3+^) was achieved via a microfluidic approach. The preparation did not require any stabilizing ligands or surfactants, minimizing potential sources of impurities. The synthesized nanomaterials were characterized from a structural (XRD and XAS at lanthanide L_3_ edges), morphological (TEM), and compositional (XPS, ICP-MS) perspective, giving complementary information on the materials’ features. In view of potential applications in the field of optical bioimaging, the optical emission properties of the doped nanoparticles were assessed, and samples showed strong luminescent properties while being less affected by self-quenching mechanisms. Furthermore, *in vitro* cytotoxicity experiments were conducted, showing no negative effects and evidencing the appeal of the synthesized materials for potential applications in the field of optical bioimaging.

## Introduction

Thanks to its sensitivity and specificity in target detection, optical bioimaging is one of the most practical approaches to molecular imaging, which has been defined as “directly or indirectly monitoring and recording the spatiotemporal distribution of molecular or cellular processes for biochemical, biological, diagnostic, or therapeutic applications”.^1^ A successful optical probe should fulfill several requirements, including excitation and emission wavelengths in the desired energy range (*vide infra*), brightness, negligible cytotoxicity, stealth properties,^2^ bio- and photostability, pharmacokinetics, and nonspecific tissue accumulation.^3^ In particular, both excitation and emission wavelengths should ideally be in the near-infrared range (NIR, typically 650–900 nm), one of the so-called “biological windows” (the second one being in the 1000–1400 nm range),^4^ which combines the virtues of good tissue penetration and low autofluorescence.^5,6^ Whereas both UV and visible light are absorbed by naturally occurring endogenous fluorophores (mostly hemoglobin and related molecules) leading to autofluorescence,^3^ UV excitation also causes ionization and tissue damage while additionally exhibiting low penetration depths.^7^

Inorganic nanoparticle-based fluorophores are particularly appealing for applications in optical bioimaging due to their notable fluorescence quantum yields, similar to most conventional organic fluorophores but with the additional advantages of higher chemical stability and tunable photoluminescence properties.^8^ Among these inorganic fluorophores, zinc sulfide, a wide-band gap semiconductor (around 3.7 eV at room temperature),^9^ is especially interesting because of its low toxicity (ZnS nanoparticles (NPs) showed an absence of cytotoxic effects on human endothelial cells even after more than six days)^10^ and thermal and bio- stability.^11,12^ ZnS NPs also have the capability to host different dopants within their structure, allowing tuning of their photoluminescence properties.

The first paper reporting ZnS doping was published in 1994 by Bhargava et al.^13^ who studied Mn-doped ZnS nanosystems, yielding both high quantum luminescence efficiency and lifetime shortening. Since then, luminescent materials particularly based on Mn-doped ZnS nanoparticles have been extensively studied^14,15^ and additionally extended to various kinds of transition-metal (TM) and rare-earth (RE) ZnS doping (TM = Cu^2+^, Fe^2+^, Pb^2+^, Ni^2+^, Cd^2+^, Co^2+^; RE = Eu^3+^, Sm^3+^, Tb^3+^, Er^3+^).^16−18^ Among these different scenarios, doping zinc sulfide with RE is particularly appealing since it allows us to achieve fluorescence in the first biological window (NIR, 650–900 nm). Moreover, it is possible to co-dope ZnS with two or more different luminescent ions, enabling multiplexed optical bioimaging.^19^

In order to apply NIR-luminescent nanosystems such as RE-doped ZnS to optical bioimaging, it is important to develop a synthetic approach providing a high degree of control on the luminescence response of the final product and consequently on the doping. Notably, the functional properties of the nanoparticles, such as photoluminescence, are strongly affected by the NP structure (crystal phase and crystallinity degree), morphology (size, shape, and anisotropy),^20^ and composition. A certain degree of control on the NPs’ final features, especially on size and shape, is commonly achieved by using ligands and/or surfactants, which hinder nuclei growth and allow us to control the final size of the products. Among these, an effective approach for the synthesis of doped ZnS nanoparticles was developed by Dolcet et al. using a two-miniemulsions technique.^17^ In such droplet-based systems, the crystallization of nanostructures takes place at room temperature in the confined space of miniemulsion droplets, limiting the growth of the nanoparticles within the individual droplet volume. Even though the use of surfactants or organic capping ligands often leads to well-dispersed nanoparticles, their removal from the NP surface is usually experimentally demanding, requiring either stripping procedures or solvent-assisted selective removal. However, such synthetic approaches can often result in impurities, detectable, for example, as carbon contamination on the NP surface by XPS or TGA.^17,21^

In order to accomplish a more sustainable water-based synthesis of functional inorganic materials, in this work, we address the synthesis of doped ZnS nanoparticles by a simple room-temperature microfluidic approach. In addition, we aim at circumventing the use of any surfactant, which would require additional purification procedures and eventually an atmosphere-controlled heat treatment to completely remove the organic impurities. We also aim to avoid high-temperature treatment since this is both energy-demanding and will likely lead to coalescence phenomena of the NPs.

In recent years, microfluidic reactors (i.e., continuous-flow reactors with micrometric-size channels, processing 10^–9^ to 10^–18^ L volumes of fluids)^22^ have become highly attractive devices for synthesizing NPs of exceptional quality.^23−26^ Microfluidic systems enable (i) the achievement of homogeneous reaction mixtures within the millisecond (ms) time scale, thanks to the rapid and continuous mixing of liquid precursors,^27^ and (ii) rapid heating and cooling of reaction mixtures by precisely and rapidly controlling the reaction temperature.^28^ This in turn ensures a high degree of control over the reaction as well as over the structural evolution of the inorganic nanocrystals. Moreover, microfluidic setups can be easily integrated with in-line detectors (e.g., based on optical spectroscopies), allowing the rapid screening of a wide range of experimental conditions and thus the optimization of synthetic parameters within short time frames.^24,25^ Thanks to these benefits, microfluidic techniques allow precise control over the final product in terms of size, size distribution, and composition.^23,29^

ZnS crystallization is a very fast process that was determined to be mass transfer-limited.^30,31^ Achieving a high degree of control over the mixing of the precursors, which is ensured in microfluidic reactors, is therefore crucial. Moreover, in continuous-flow conditions, a temporal separation of the NP nucleation and growth phases is pursued, leading to a small NP size distribution and uniform morphology.^28^

The first study reporting the microfluidic synthesis of undoped ZnS nanoparticles without capping agents or surfactants was performed by Dengo et al.,^32^ who optimized a synthetic approach, in particular, in terms of size and size distribution of the obtained NPs using earth-abundant and cheap precursors such as zinc nitrate and sodium sulfide. In the present work, we extend this approach to the continuous-flow, ligand-free synthesis of small doped zinc sulfide nanoparticles. In this way, we aimed at tuning the luminescent properties of the nanoparticles. First, we synthesized Mn-doped ZnS nanoparticles via our microfluidic approach as a proof of concept of the method. Subsequently, zinc sulfide was doped with luminescent lanthanide ions in order to achieve photoluminescence in the NIR range, which is important for *in vivo* optical bioimaging applications.

Herein, we report the synthesis of zinc sulfide NPs doped with Mn^2+^, Eu^3+^, Nd^3+^, Sm^3+^, and Yb^3+^ (at atomic percentages of 0.1, 1, and 5% with respect to the total amount of metals Zn + M) via a simple, green, and room-temperature microfluidic approach. The resulting powders were characterized from a structural, morphological, and compositional point of view, and their luminescence properties were explored. Furthermore, the local structure around Eu^3+^ and Nd^3+^ in ZnS:Eu and ZnS:Nd was elucidated by means of X-ray absorption spectroscopy (XAS). Finally, the cytotoxicity of the nanoparticles was investigated in view of possible applications in the optical bioimaging field.

## Results and Discussion

Using a microfluidic approach to achieve optimal mixing of the precursor solutions, we pursued the controlled formation of highly crystalline doped ZnS nanoparticles. Several dopants were added to the precursor feed, but the most promising results were achieved with the samples doped with Mn^2+^, Eu^3+^, and Nd^3+^. Therefore, only the Mn-, Eu-, and Nd-doped samples are discussed in detail, while further samples (doped with Sm and Yb) are presented in the Supporting Information.

### Crystal Structure and Dimensions

**Figure 1:**
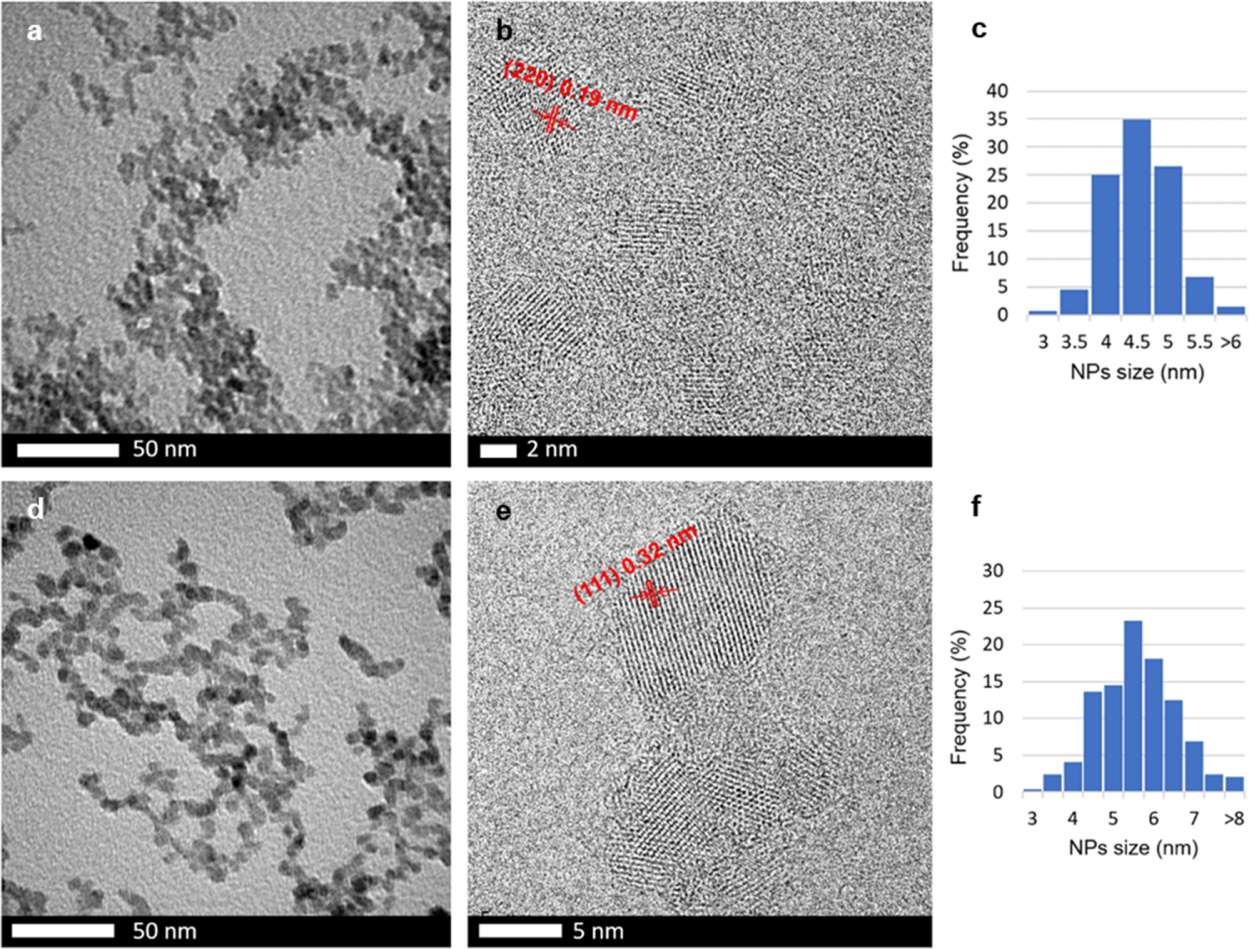
TEM and HRTEM micrographs of undoped (a, b) and 5 at. % Eu-doped (d, e) zinc sulfide NPs and relative size distributions (c and f, respectively). Lattice spacings of (111) and (220) planes of cubic ZnS are highlighted in red.

The obtained NP powders were first characterized by X-ray powder diffraction (XRPD), which confirmed the formation of ZnS nanocrystals with a cubic sphalerite structure (ICSD no. 00-005-0566). Sphalerite or zinc blende is the thermodynamically stable phase of zinc sulfide at room temperature (for bulk ZnS above 1020 °C, the stable form is wurtzite).^33^ By whole-powder pattern fitting (WPPF) of the diffractogram,^34^ an average ZnS crystallite size of 4.6 ± 0.1 nm was evaluated (fitting of undoped ZnS diffractogram is shown in Figure S1 and the corresponding refined parameters in Table S1). Bright-field transmission electron microscopy (BF-TEM) micrographs (Figure 1a) further evidenced the presence of rather monodispersed nanoparticles with an average size of 4.8 ± 0.5 nm (see size distribution in Figure 1c), thus confirming the XRPD results. Moreover, high-resolution TEM (HRTEM) micrographs (Figure 1b and others in Figure S14) showed lattice spacings of 0.19 and 0.31 nm that were assigned to (220) and (111) planes of the cubic pattern of ZnS, respectively, in agreement with XRPD results. Thus, HRTEM micrographs demonstrated that every single NP behaves as an individual coherently scattering domain. The crystallites are not further organized in larger aggregates but are constituted by single nanoparticles, retaining their own identity and shape. This evidence confirms that, under our microfluidic conditions, the aggregation and/or ripening of nanoparticles is very limited. It is worth reiterating that such small nanoparticles were obtained without the use of any ligands and/or surfactants to control nuclei growth and the final size of the NPs. This is a remarkable result as we recently reported for the first time, to the best of our knowledge, a surfactant-free synthesis of zinc sulfide by microfluidics.^35^ Indeed, only one microfluidic synthesis of undoped and Mn-doped ZnS nanoparticles was retrieved in the literature, and it employed thioglycerol as a capping agent.^36^

Our significant results could be linked to the unique conditions that are achieved in a microfluidic reactor and can be explained by the mechanism of NP formation proposed by LaMer and Dinegar.^37,38^ According to this model and, in particular, its implementations by Sugimoto^39,40^ and subsequently by Chu et al.,^41^ the mechanism of NP formation occurs through four distinct steps: (i) formation of a supersaturated solution, (ii) nucleation, (iii) growth, and (iv) aggregation of the nuclei. Once the solute concentration exceeds the supersaturation limit, the formation of many nuclei occurs in a short burst (“LaMer burst”) and the solute concentration decreases below the critical nucleation concentration, thus hindering any further nucleation and freezing the number of nuclei formed. Then, the overall free energy of the system (particles and solutes) is lowered by particle growth, occurring until all free solute has been consumed. In the absence of an effective stabilization, another way of lowering the free energy of the system consists in the aggregation of individual nanoparticles. In order to obtain small monodisperse NPs, growth should stop when particles are still in the desired nanometer scale range, for instance, due to reactant depletion, and NP aggregation should be avoided. Under the unique conditions of a microfluidic reactor, this can be achieved thanks to several advantages including (i) highly efficient and fast mixing and (ii) the flowing and dynamic nature of the system. First, the small dimensions of the tubing (diameter of few hundreds of micrometers) and the controlled mixing of precursors give rise to a highly homogeneous chemical environment in terms of the reactant concentration and temperature. Under such conditions, random (both timely and spatially) nucleation bursts and growth by uncontrolled agglomeration can be avoided. Then, the continuous-flow nature of the synthetic approach allows a temporal (and consequently spatial) separation of the NP nucleation and growth stages. Moreover, since the reactant feeding is dynamic, the solute concentration can be accurately controlled, even spatially. It follows that, if after a certain residence time (i.e., after a certain tubing length), a quenching step takes place, growth through aggregation and/or ripening phenomena is minimized and small and monodispersed NPs can be obtained.

**Figure 2:**
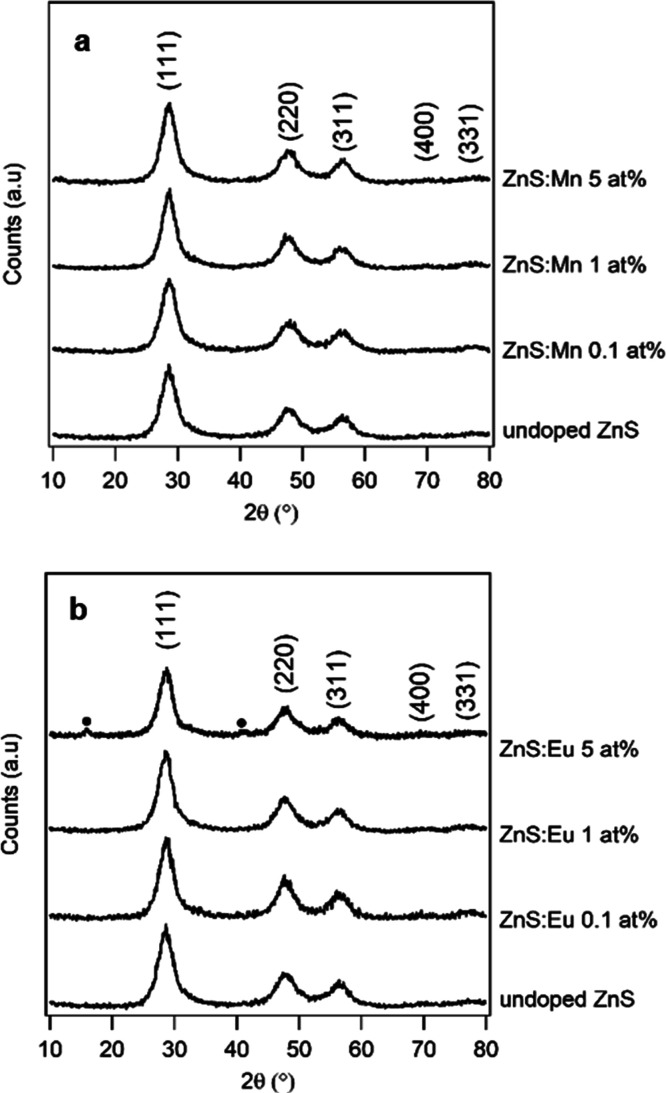
Comparison of diffractograms of undoped and (a) Mn-doped and (b) Eu-doped ZnS samples at different atomic percentages. Eu(OH)_3_ reflections are highlighted by a solid circle.

When introducing Mn^2+^, Eu^3+^, Sm^3+^, Nd^3+^, and Yb^3+^ as dopant ions, the cubic crystalline structure of zinc sulfide was retained, as evidenced by XRPD. Comparisons between the diffractograms collected from undoped, Mn-doped, and Eu-doped ZnS samples (as an example of Ln-doped samples) at different atomic percentages are shown in Figure 2a,b, respectively. Comparisons between XRPD patterns of undoped and Ln-doped samples other than Eu (Ln = Nd, Sm, Yb) at different atomic percentages are reported in the Supporting Information (Figures S12 and S13). As can be seen, while in the case of Mn-doped samples, no other phases outside of sphalerite were detected, diffractograms of lanthanide-doped samples with atomic doping percentages of 5% showed the presence of Ln(OH)_3_ in addition to sphalerite, with the exception of the Yb-doped sample. The precipitation of lanthanide hydroxides together with ZnS at high Ln^3+^ concentrations can be rationalized considering the basic pH of the reaction mixture (as sodium sulfide was used as a sulfur precursor) and the well-known oxophilicity of lanthanides.^42^ The Ln(OH)_3_ content in the 5 at. % doped samples was estimated to be between 1.5 and 3.6 wt % of the total mass by WPPF (fittings of doped ZnS XRPD patterns are reported in Figures S2, S4, S6, S8, and S10; corresponding refined parameters in Tables S2–S6). As expected, an increase in the lattice parameter *a* upon doping was observed, indicating a very slight expansion of the sphalerite unit cell when introducing dopants (Figure S3, S5, S7, S9, and S11). However, no trend as a function of the dopant concentration was found. In addition, since the reflections were very broad, no clear shift in the position of reflections was observed between doped and undoped samples. The average crystallite size of sphalerite in the doped samples was estimated by WPPF to range from 4.8 to 6.2 nm (see the Supporting Information, Tables S2–S6) and, as well as for undoped sample, this result was confirmed by TEM micrographs, showing quite monodispersed NPs with average sizes of 5.4–5.8 nm (see 5 at. % Eu-doped NPs TEM micrograph in Figure 1d and others in the Supporting Information, Figures S15, S16, and S18–S20). In addition, HRTEM micrographs of the 5 at. % Eu-doped sample (Figure 1e and others in Figure S17) showed that, notably, when introducing dopants, the synthetic output always comprises single and independent nanoparticles. Moreover, the lattice spacing of (111) and (220) planes of ZnS:Eu 5 at. % obtained from HRTEM analysis did not show detectable changes compared to the undoped sample (Figure 1b and Figure S14). In Table 1, a comparison between NP sizes evaluated from XRPD fitting and TEM of undoped and 5 at. % doped ZnS samples is reported. Remarkably, within experimental uncertainties, the sizes determined via XRPD diffractogram fittings are comparable with TEM results, confirming that primary NPs are obtained and no aggregation occurs. Moreover, it can be observed that doped ZnS NPs are slightly larger than undoped ones, but the control over the NP size and size distribution enabled by the microfluidic approach was confirmed to be effective in both cases.

**Table 1:** Comparison between Average Crystallite Size Obtained from XRPD Fitting and Average NP Sizes Obtained from TEM Micrographs for Undoped and 5 at. % Doped ZnS Samples

sample	average crystallite size (nm) from XRPD	average NP size (nm) from TEM
undoped ZnS	4.6 ± 0.1	4.8 ± 0.5
ZnS:Mn 5 at. %	4.8 ± 0.1	5.5 ± 0.9
ZnS:Eu 5 at. %	5.1 ± 0.1	5.8 ± 1.0
ZnS:Nd 5 at. %	5.5 ± 0.1	5.6 ± 1.0
ZnS:Sm 5 at. %	6.2 ± 0.1	5.5 ± 0.9
ZnS:Yb 5 at. %	4.9 ± 0.1	5.4 ± 0.7

The formation and stability of the obtained ZnS NP suspension in water were investigated by Dengo et al. in a previous work.^32^ There, it was found that, during the product workup, an optically transparent and opalescent suspension was formed, which did not change from visual inspection over several weeks. The ζ potential on the suspension at autogenous pH (10–12) was determined to be −40 mV.^32^

### Surface and Bulk Composition

**Figure 3:**
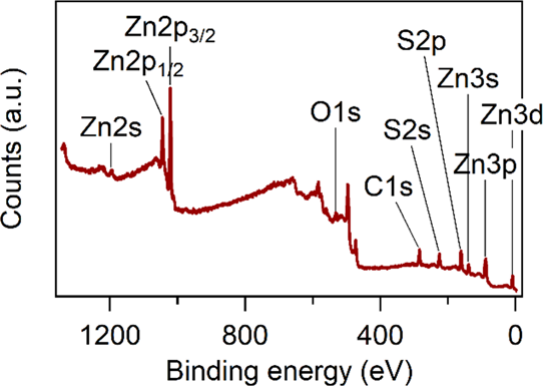
XPS survey spectrum of undoped ZnS. Binding energy is corrected for charge effects.

Since the interaction between the metal sulfide nanostructures and their physiological environment occurs at the surface, an extensive characterization of their surface chemistry is essential in view of applications in optical bioimaging. In this regard, the surface composition of undoped and 5 at. % doped NPs was analyzed by X-ray photoelectron spectroscopy (XPS). A representative survey spectrum (0–1350 eV) collected from undoped ZnS is reported in Figure 3, showing the presence of zinc and sulfur as well as oxygen and carbon. The latter two were present due to unavoidable adventitious contaminations during sample handling.^43^ The effectiveness of the sample purification protocol was proven by the absence of Na and N signals (expected at 1072 and about 400 eV, respectively),^44,45^ which could be present as impurities from precursor counterions. XPS semi-quantitative analysis showed that the sample surfaces were slightly enriched in Zn with an average Zn:S atomic ratio of 1:0.7 (see the Supporting Information, Table S7, for detailed surface composition data of 5 at. % doped samples). The formation of a metal-rich surface is commonly observed for the synthesis of metal chalcogenides in aqueous suspensions.^46^

**Figure 4:**
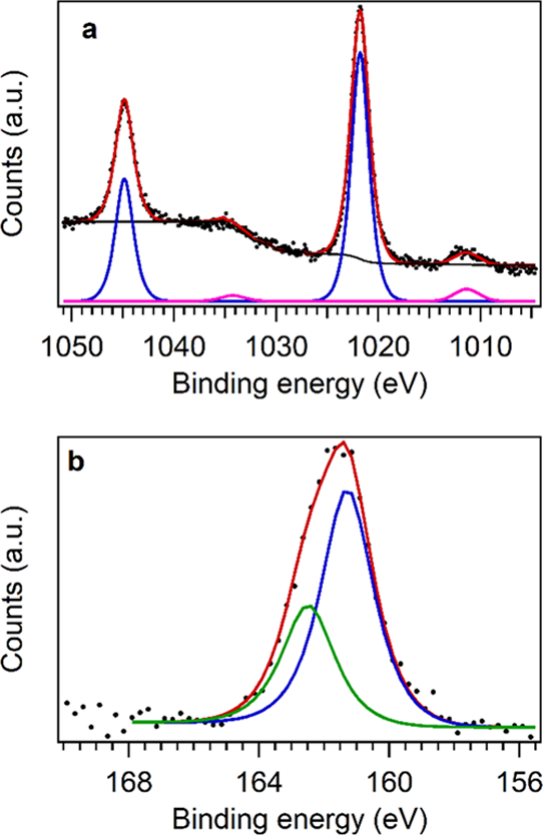
Fitting (red lines) of (a) Zn2p photoemission peaks (blue line: photoemission peaks and pink line: shake up satellite peaks) and (b) the S2p peak (blue line: S2p_3/2_ component and green line: S2p_1/2_ component, separated for clarity) of ZnS:Nd 5 at. %. Sulfate species would be expected at 169 eV. BE values are corrected for charge effects.

The Zn2p_3/2_ signal was found for all samples at a binding energy (BE) of 1021.6–1021.9 eV, whereas the S2p signal was in the 161.2–161.6 eV range (detailed BEs are reported in Table 2). Both values are compatible with literature reports for ZnS (Zn2p_3/2_ = 1022.0 eV; S2p = 161.6 eV).^17,18,21,45,47^ Since it is not possible to discriminate between zinc sulfide and zinc oxide from the position of the Zn2p_3/2_ peak (a representative fitting is shown in Figure 4a; the others are reported in Supporting Information, Figures S21a–S25a), the modified Auger parameter (AP, α = BE_Zn2p_3/2__ + KE_ZnLMM_, where KE is the kinetic energy of the peak)^48^ was calculated. The resulting APs (2011.1–2011.5 eV, detailed values in Table 2) confirmed the presence of ZnS (AP = 2011.5 eV)^49^ instead of ZnO (AP = 2009.5–2010.3 eV)^50^ on all sample surfaces. Moreover, the S2p signal fittings (a representative one is shown in Figure 4b; the others are reported in Supporting Information, Figures S21b–25b) showed the exclusive presence of sulfide on the NP surface, ruling out the presence of SO_4_^2–^ groups (a typical signal at a BE around 169 eV),^45^ which could be present due to partial superficial oxidation commonly found in sulfides.^42,51^

**Table 2:** Binding Energy (BE) and Auger Parameter (AP) Values

sample	BE Zn2p_3/2_ (eV)	AP (eV)	BE S2p (eV)
ZnS	1021.7	2011.1	161.4
ZnS:Mn 5 at. %	1021.9	2011.1	161.6
ZnS:Eu 5 at. %	1021.6	2011.4	161.2
ZnS:Nd 5 at. %	1021.8	n.a.	161.4

Dopant signals could not be detected in any of the samples, even in the ones featuring higher dopant concentrations (5 at. %). The absence of signals regardless of the dopant concentration allowed us to exclude the segregation of dopants to the NP surface, thus indicating that dopants were included in the zinc sulfide matrix.

The presence of the dopants in the final materials was nevertheless confirmed by inductively coupled plasma-mass spectrometry (ICP-MS) measurements (Table 3). The incorporation of dopants in the final products is quantitative in nearly all samples (i.e., the experimental dopant content is comparable to nominal content), confirming that they are included within the sample bulk. In the case of Nd-doped samples, an experimental doping percentage lower than the nominal one was found. This might be due to only partial precipitation of insoluble neodymium hydroxide (*vide infra*) with soluble species washed away during the purification phase. It is worth noting that the incorporation of lanthanide ions into the zinc sulfide structure is particularly challenging due to their trivalent charge (compared to the divalent zinc), their much larger ionic radius with respect to Zn^2+^ ions (*r*_Zn(II)_ = 60 pm, *r*_Eu(III)_ = 94.7 pm, and *r*_Nd(III)_ = 98.3 pm),^52^ and their well-known oxophilic behavior. On the other hand, the retrieved experimental atomic percentage higher than the nominal atomic percentage found for the 5 at. % Mn and Eu-doped samples could be due to the loss of zinc during the washing step. It should be noted that the percentage of doping was defined as the ratio between the dopant content and the content of zinc plus dopant. A higher experimental doping percentage than expected could therefore result from the loss of zinc.

**Table 3:** ICP-MS Measurements (Relative Error: ± 5%)

	atomic percentage	
	nominal	experimental
ZnS:Mn	0.1	0.10
	1	1.06
	5	5.96
ZnS:Eu	0.1	0.07
	1	0.90
	5	6.08
ZnS:Nd	0.1	0.08
	1	0.48
	5	2.78

### X-ray Absorption Spectroscopy

**Figure 5:**
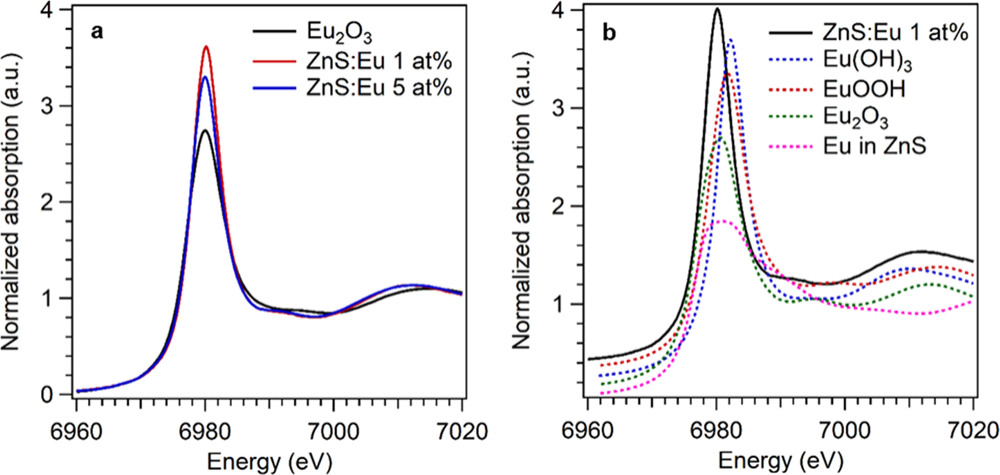
Eu L_3_ edge XANES spectra for Eu-doped samples, compared to (a) reference oxide and (b) the spectrum of the 1 at. % doped sample compared with calculated theoretical references (spectra shifted vertically for clarity).

**Figure 6:**
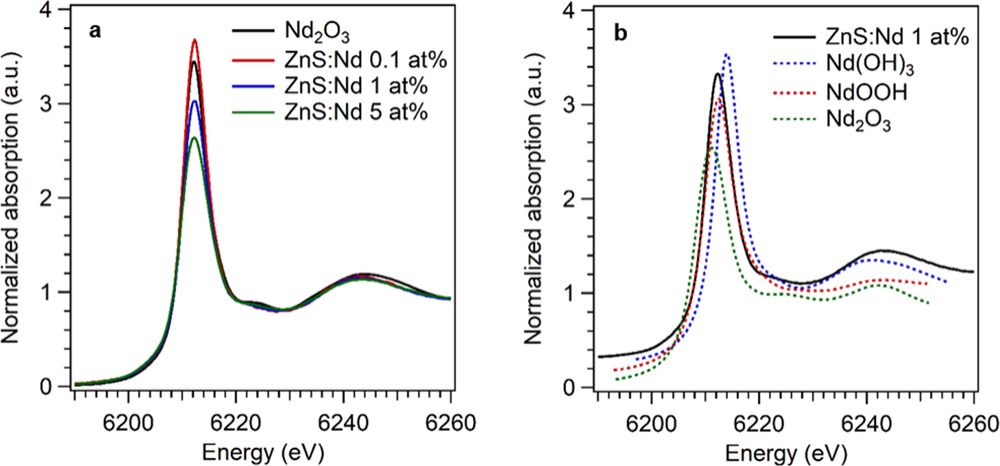
(a) Nd L_3_ edge XANES spectra for Nd-doped samples acquired in fluorescence mode and (b) the spectrum of the 1 at. % doped sample compared with calculated theoretical references (spectra shifted vertically for clarity).

Although the 5 at. % lanthanide-doped samples showed the presence of Ln(OH)_3_, at lower doping levels it was not clear whether the same species formed or whether instead the dopants were hosted in the sphalerite matrix. To elucidate the structure around the dopant, we performed X-ray absorption spectroscopy (XAS) experiments at the Eu and Nd L_3_ edge. At the Eu L_3_ edge, spectra were collected at intermediate (1 at. %) and high (5 at. %) Eu contents, while at the Nd edge, the low doping level (0.1 at. %) was also investigated through measurements in the fluorescence mode. The recorded X-ray absorption near-edge structure (XANES) spectra for the experimental samples doped with Eu or Nd are reported in Figures 5a and 6a, respectively.

The experimental spectra for the Eu-doped samples show an intense white line at around 6980 eV. The peak position is similar to the reference europium(III) oxide, indicating that the dopant is indeed in a trivalent oxidation state, but the other features of the two spectra are different. Eu_2_O_3_ shows a less intense white line and two features after the edge at about 6995 and 7014.6 eV. For the Eu-doped ZnS samples, similar features can also be found but at 6992.8 and 7012.5 eV. These differences in the white line intensity and feature positions indicate that the structure around the Eu ions is indeed different from the oxide and with an increased number of coordinated atoms. This is also confirmed by the comparison between the ZnS:Eu 1 at. % sample and calculated references (Figure 5b). Several possible structures were considered, in particular, a Eu^2+^ ion (to maintain charge balance) substituting Zn in the sphalerite lattice (coordination number CN: 4), Eu_2_O_3_ (CN: 6), EuOOH (CN: 7), and Eu(OH)_3_ (CN: 9). As can be seen in Figure 5b, with an increasing coordination number, there is a clear sharpening of the white line. This trend indicates that the expected coordination number around the Eu ions in the experimental samples is therefore greater than the reference oxide. In addition, the spectral features of the experimental curves fit well with those of europium hydroxide, even if exact positions of the features do not match perfectly with those of the calculated spectra.

Analogous results also hold for the Nd-doped samples where the XANES data at the Nd L_3_ edge is shown in Figure 6. The formation of structures resembling that of the hydroxide around the neodymium ions is also confirmed by the comparison with the calculated spectra (in Figure 6b, the simulated spectra of Nd_2_O_3_, Nd(OH)_3_, and NdOOH are reported). Furthermore, with increasing Nd content, the white line intensity decreases and, in the 5 at. % sample, a shoulder at 6224.5 eV grows. This indicates the coexistence at high Nd concentrations of both Nd(OH)_3_ and Nd_2_O_3_ moieties.

To confirm these hypotheses, a first-shell fitting of the EXAFS data was carried out for all the samples (see the Supporting Information, Figures S26 and S27). Additionally, models including a sulfur-only or a mixed sulfur-oxygen first shell were considered during the fitting, but these models could not reproduce the features in a satisfactory way. This indicates that no Ln–S interaction is occurring, confirming the oxophilic behavior of the lanthanide ions. The results derived from the extended X-ray absorption fine structure (EXAFS) analyses are summarized in Table 4. The coordination number for the Eu-doped samples is close to 9 for both samples with the Eu–O distance increasing with the doping level toward the values of bulk crystalline Eu(OH)_3_.

**Table 4:** Results of the EXAFS Fitting Procedure of the EXAFS Curves for Eu- and Nd-Doped Samples

sample	shell	N	distance (Å)	*D* cryst Eu(OH)_3_ (Å)	σ^2^ (10^–3^ Å^2^)	*E*_0_ (eV)	*R* factor
Eu L_3_ edge							
ZnS:Eu 1 at. %	Eu-O	8.9 ± 1.6	2.42 ± 0.02	2.47	8.7 ± 3.6	5.7 ± 1.7	1.4%
ZnS:Eu 5 at. %	Eu-O	9.4 ± 1.5	2.44 ± 0.02	2.47	10.7 ± 3.3	6.5 ± 1.4	1.0%
Nd L_3_ edge							
ZnS:Nd 0.1 at %	Nd-O	8.6 ± 1.9	2.50 ± 0.02	2.54	9.7 ± 4.3	2.7 ± 1.9	1.9%
ZnS:Nd 1 at %	Nd-O	8.4 ± 1.9	2.51 ± 0.03	2.54	8.7 ± 4.5	2.8 ± 2.1	2.5%
ZnS:Nd 5 at %	Nd-O	8.0 ± 1.4	2.50 ± 0.02	2.54	7.8 ± 3.6	2.9 ± 1.6	2.0%

At a low Nd concentration, the Nd–O coordination number is close to 9, as in the hydroxide, but as the content increases, the CN reduces and also the Nd–O distance remains intermediate between that of the hydroxide (Nd–O = 2.54 Å) and the oxide (Nd–O = 2.47 Å).

A comprehensive consideration of these results as a whole indicates that, even though the dopants are incorporated in the samples, as confirmed by ICP-MS, the ions are not hosted within the sulfide matrix but rather form separate phases.

### Absorption and Photoluminescence Properties

**Figure 7:**
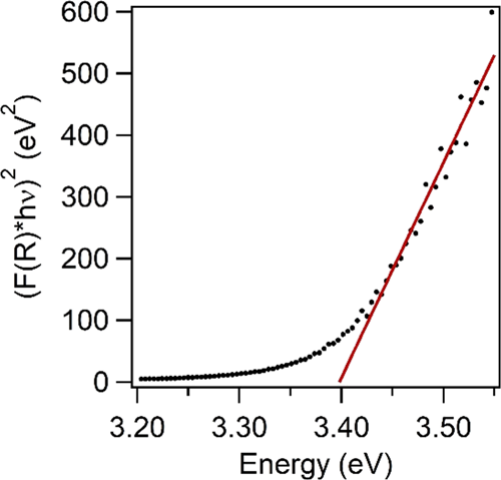
Tauc plot ((*F*(*R*)*hv*)^2^ vs *hv*) of the undoped ZnS sample.

Since our synthesized doped nanoparticles could be employed in the optical bioimaging field as fluorescent probes, their photoluminescence properties were assessed. As a first step, the optical absorption properties of the undoped ZnS NPs were explored using the diffuse reflectance technique (Figure S28). The optical band gap (*E*_g_) can be determined using the following relation^53^
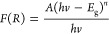
where *F*(*R*) is the diffuse reflectance, *n* is a constant equal to 1/2 for a direct band gap semiconductor such as ZnS, and *A* is a proportionality constant. *E*_g_ can be estimated from a plot of (*F*(*R*)*hv*)^2^ versus *hv* (“Tauc plot”, Figure 7) by extrapolating the straight portion to the energy axis at *F*(*R*) = 0. *E*_g_ was found to be 3.40(5) eV, corresponding to a wavelength of 365 nm. The obtained *E*_g_ is similar to that found for bulk ZnS by Kurnia et al. (around 3.6 eV).^54^ It has to be noted that the actual sizes of the present NPs (around 5–6 nm, see Table 1) is much higher than the exciton Bohr radius for ZnS (2.5 nm)^11^ and therefore the quantum confinement effect is negligible in line with the found energy bandgap value. On the other hand, the emission of the undoped sample upon UV excitation in the bandgap region (excitation around 365 nm) is barely detectable as it can be noted from the picture shown in the Supporting Information (Figure S28) where a comparison among the ZnS NPs samples is shown upon excitation with a Wood’s lamp.

**Figure 8:**
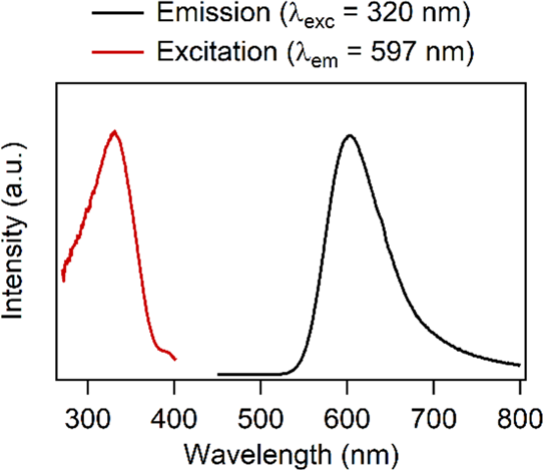
Emission spectrum (black line, λ_exc_ = 320 nm) and excitation spectrum (red line, λ_em_ = 597 nm) of the 5 at. % Mn-doped ZnS nanoparticles.

The Mn^2+^ doped samples show a bright orange emission when excited in the UV region. The excitation spectrum of the 5 at. % doped ZnS:Mn sample (shown as a red line in Figure 8) evidenced a broad band in the 280–380 nm UV region, showing a maximum value around 330 nm, corresponding to the band edge transition in agreement with other authors.^55,56^ This behavior is typical of Mn^2+^-doped ZnS, and it is very similar to the ones obtained by other authors on the same compound^57^ due to bandgap transitions. Indeed, the absorption band corresponding to band-to-band transition for the undoped ZnS NPs perfectly overlaps with the excitation band around 330 nm observed for the Mn-doped samples (see below and Figure S30), thus confirming the hypothesis of bandgap transitions as responsible for the Mn^2+^ excitation.

In agreement with literature data, Mn-doped ZnS nanoparticles showed a strong emission in the orange region, extending from 525 to 700 nm (shown in Figure 8 for the 5 at. % doped sample), which can be attributed to the ^4^T_1_ → ^6^A_1_ transition of Mn^2+^ ions.^58^ In order to show at a glance the relative brightness of the differently doped ZnS:Mn samples, we prepared a series of quartz cuvettes filled with the undoped and Mn-doped samples at different doping percentages and illuminated them with a Wood’s lamp (λ = 365 nm). Figure S29 depicts samples under simultaneous illumination with UV radiation. From this image, it can be observed that the undoped sample has a negligible emission in the visible region, while the brightest sample corresponds to the NPs with a higher dopant concentration. This behavior is in line with that reported by Meijerink et al. for similar ZnS:Mn samples^56,59^ and by other groups.^60^

**Figure 9:**
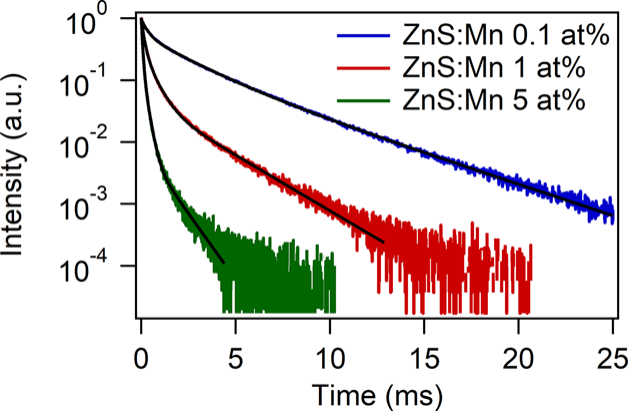
Emission decay curves for 0.1 at. % (blue line), 1 at. % (red line), and 5 at. % (green line) Mn-doped ZnS nanoparticles. Black line: multi-exponential fitting.

To shed light on the excited-state dynamics, the emission decays of differently doped ZnS:Mn nanoparticles were measured (Figure 9) and they appear to strongly shorten on increasing the Mn^2+^ concentration in the ZnS host. The decay of the luminescence in the red region (around 600 nm) of the Mn^2+^ ions in doped nanocrystalline ZnS was investigated in detail by some authors (as for instance Bol and Meijerink^56^ and Zheng et al.^61^), who found a multiexponential behavior for the emission decay curve. In particular, they observed fast decays of the order of some tenths or even hundreds of microseconds and a slow component in the millisecond regime. According to Zheng et al.,^61^ we fitted the emission decays using a triexponential function:
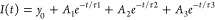
where τ_1_, τ_2_, and τ_3_ are the time constants and *A*_1_, *A*_2_, and *A*_3_ are normalized amplitudes of the components.

The time constants, amplitudes, and average lifetimes obtained from the fittings are reported in Table 5. The slowest decay can be attributed to the ^4^T_1_ → ^6^A_1_ transition of isolated Mn^2+^ ions in the ZnS crystalline host. On the other hand, the fastest components can be attributed to exchange coupled Mn^2+^ ion pairs.^62^ These fast components could also be due to emission of Mn^2+^ ions with an enhanced overlap between the 3d and sp host states caused by lattice strain,^63^ which is well present in nanocrystalline hosts that have a high surface to volume ratio and therefore relevant lattice strain at the surface (see Tables S1–S6 for estimated microstrain values). From the decay curves (Figure 9) and from the values obtained from the fit (Table 5), a strong decrease of the lifetimes on increasing the Mn^2+^ concentration in the ZnS host is observed. Indeed, the average lifetime was found to be 3.09 ms for the sample with the lowest Mn concentration, decreasing to 0.20 ms for the one with a higher doping percentage. This behavior is in perfect agreement with the trend found by Park et al.^63^ for Mn^2+^-doped ZnS thin films and also Chen et al.^55^ for Mn-doped ZnS NPs, who evidenced a strong emission concentration quenching even at quite low Mn^2+^ doping percentages (2–3 mol %). Remarkably, the average decay time for the ^4^T_1_ energy level of Mn^2+^ for the sample with a lower dopant concentration (0.1 at. %) is 3.09 ms. This value is much longer than the one found for bulk ZnS:Mn^2+^ (1.8 ms, according to Gumlich),^64^ confirming the high crystallinity of the samples.

**Table 5:** Decay Lifetimes τ, Normalized Amplitudes *A*, and Average Lifetimes τ_av_ Obtained from the Fitting of the Emission Decay Curves for Mn-Doped Samples

Mn^2+^ concentration	τ_1_ (ms)	*A*_1_ (%)	τ_2_ (ms)	*A*_2_ (%)	τ_3_ (ms)	*A*_3_ (%)	τ_av_ (ms)
0.1 at. %	4.30 ± 0.02	22	2.00 ± 0.02	35	0.330 ± 0.005	42	3.09 ± 0.03
1 at. %	2.41 ± 0.01	5	0.536 ± 0.006	30	0.134 ± 0.002	65	1.04 ± 0.02
5 at. %	1.02 ± 0.02	1	0.206 ± 0.002	20	0.059 ± 0.001	79	0.201 ± 0.004

It is worth mentioning that the 0.1 at. % Mn-doped sample shows an emission decay that is among the slowest evidenced in the literature for ZnS:Mn nanocrystalline samples,^60,65^ and therefore, it is interesting to consider for applications in which a long fluorescence lifetime is needed, such as fluorescence lifetime imaging (FLIM).^66^ Moreover, the strong decrease of the luminescent lifetime with an increasing concentration of dopants confirms that a self-quenching mechanism involving Mn^2+^ ions is present.

**Figure 10:**
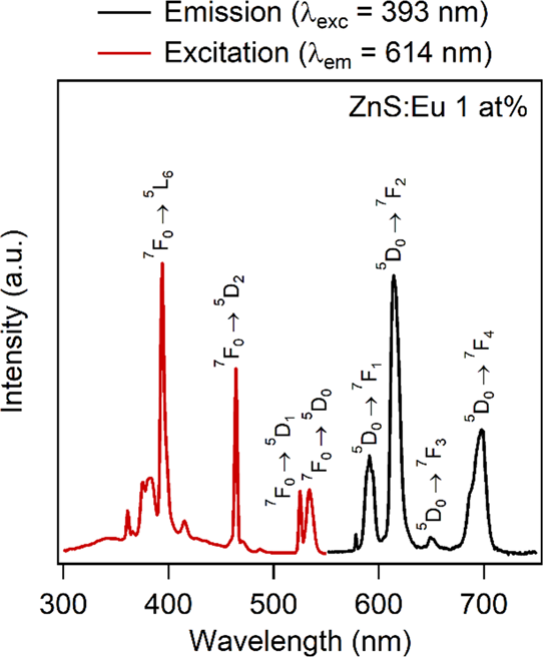
Emission spectrum (black line, λ_exc_ = 393 nm) and excitation spectrum (red line, λ_em_ = 614 nm) of the 1 at. % Eu^3+^-doped ZnS nanoparticles.

Since the luminescence properties of the Mn-doped samples showed the typical features of substitutional doping, we also studied the luminescence properties of the Ln-doped samples to compare their response. The emission spectrum of Eu-doped ZnS nanoparticles displayed different behaviors for different dopant concentrations. The emission bands of ZnS:Eu 1 at. % (Figure 10) are typical of the Eu^3+^ ions with the most intense band around 614 nm due to the hypersensitive ^5^D_0_ → ^7^F_2_ transition. We calculated the asymmetry ratio (defined as the ratio between ^5^D_0_ → ^7^F_2_/^5^D_0_ → ^7^F_1_ emission transitions), which provides information about the average symmetry around the Eu^3+^ ions. This ratio was estimated to be around 3, evidencing a significantly distorted local environment around the lanthanide ion^67^ as also evidenced by the detailed EXAFS analysis (Table 4). This is a reasonable behavior due to the notable difference between the ionic radii of the Zn^2+^ ions (0.6 Å in a fourfold coordination)^52^ and the Eu^3+^ ions (1.07 Å in a ninefold coordination).^52^ Moreover, the widths of the emission bands, which are very broad, also evidenced a high degree of local disorder. On the other hand, the ZnS:Eu 5 at. % sample under a 393 nm excitation exhibited emission bands (see the Supporting Information, Figure S31) attributable to segregated trivalent europium hydroxide, confirming what was also evidenced by XRPD (see Figure 2b). The excitation spectrum of the Eu-doped sample shows the typical features due to absorption from the ^7^D_0_ state with relatively sharp bands typical of lanthanide transitions.

Moreover, in the UV region, a large excitation band in the 300–350 nm range with a plateau maximum around 330–340 nm was observed. The overlapping of this band with the sharp bands typical of the Eu^3+^ ions is significant as its signal-to-noise ratio is similar to the excitation band due to the ZnS host shown in Figure 8. Therefore, a direct sensitization of the Eu^3+^ ions by the ZnS host is present. This behavior has been also found by Mukherjee et al.,^68^ who proposed that ZnS:Eu^3+^ can act as a potential electron trap and the sensitization can be achieved either by direct bandgap excitation or by a valence band-to-Eu^2+^ transition, both mechanisms explaining the experimental findings.

**Figure 11:**
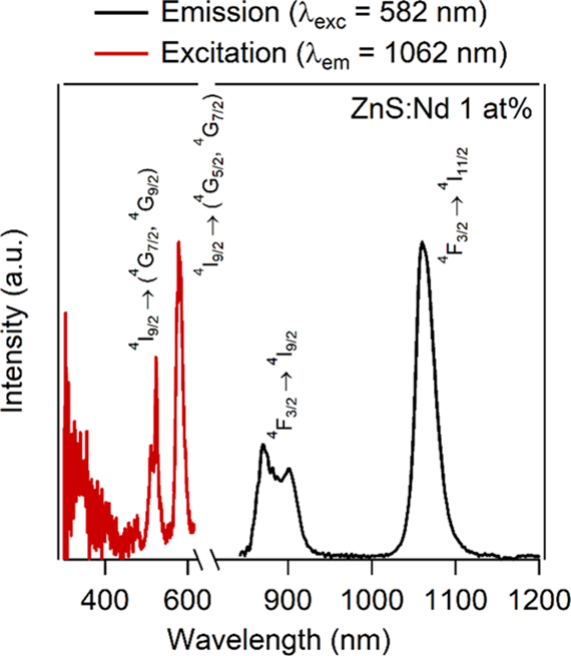
Emission spectrum (black line, λ_exc_ = 582 nm) and excitation spectrum (red line, λ_em_ = 1062 nm) of the 1 at. % Nd^3+^-doped ZnS nanoparticles.

Concerning Nd-doped samples, the excitation spectrum (red line in Figure 11) of ZnS:Nd 1 at. % featured bands due to transitions attributed to Nd^3+^ ions. In particular, the excitation band around 525 nm can be attributed to the ^4^I_9/2_ → (^4^G_7/2_, ^4^G_9/2_) transitions, while the one around 580 nm can be assigned to the ^4^I_9/2_ → (^4^G_5/2_, ^2^G_7/2_) transitions.^69^ As in the case of the Eu-doped ZnS sample, in the 300–350 nm range of the UV region, a large excitation band with an intensity maximum of approximately 330–340 nm is observed. Similarly, this band in the UV region is significant, and therefore a direct sensitization of the Nd^3+^ ions by the ZnS host is present, explaining the origin of the observed excitation band. On the other hand, after excitation at 582 nm, strong emission bands in the near-infrared region (black line in Figure 11) were observed, one around 900 nm (^4^F_3/2_ → ^4^I_9/2_)^69^ and another around 1065 nm (^4^F_3/2_ → ^4^I_11/2_),^69^ suggesting that the studied nanomaterials may be suitable for applications in nanomedicine in the near-infrared biological window.

### *In Vitro* Cytotoxicity

**Figure 12:**
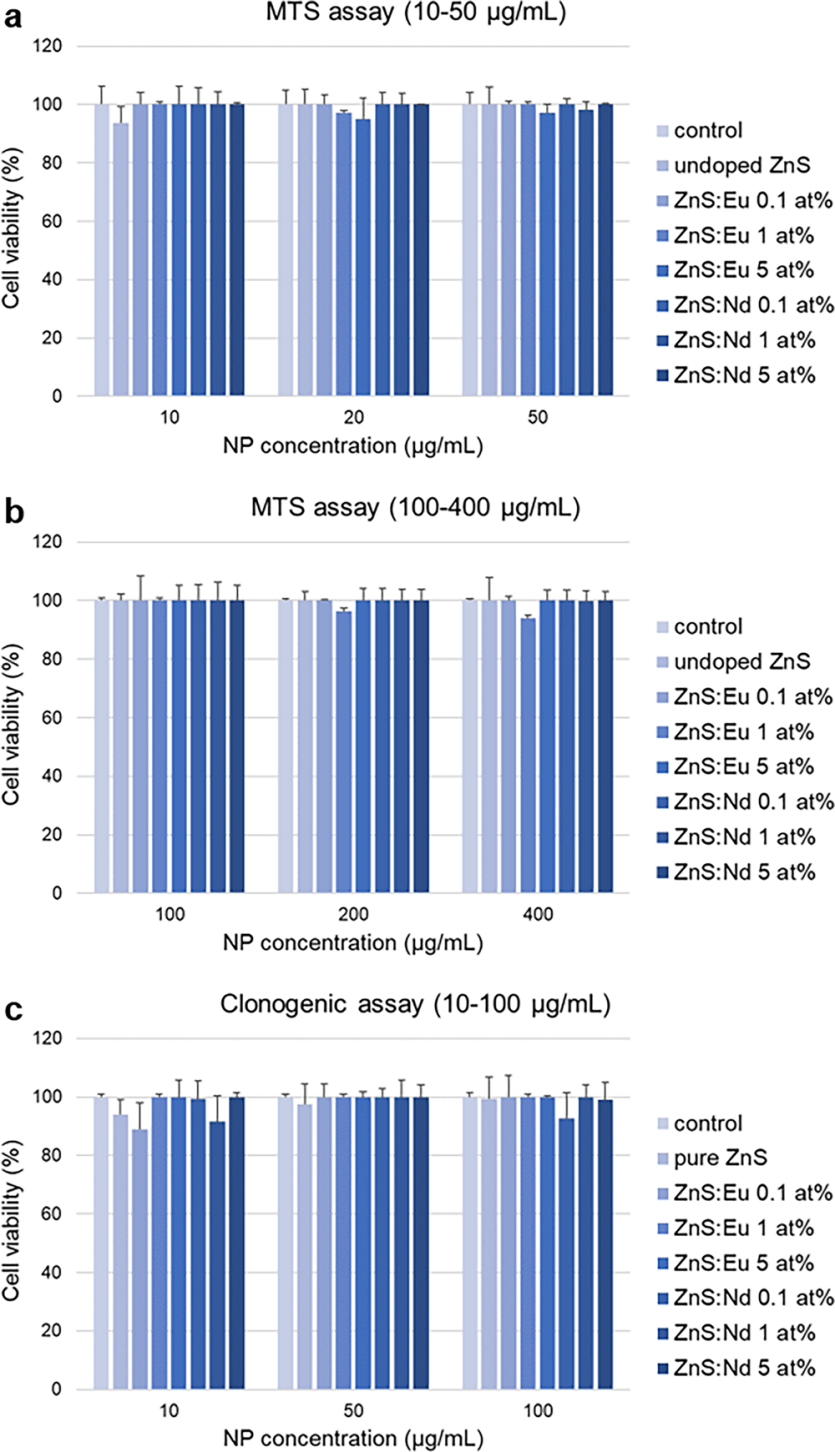
Cytotoxicity of ZnS NPs toward human A549 alveolar carcinoma cells. Cell viability was determined by an MTS assay (NP concentrations of (a) 10–50 μg/mL and (b) 100–400 μg/mL) and (c) clonogenic assay at the end of a 24 h incubation in media containing different concentration of ZnS NPs. Data are means ± S.D. from independent experiments performed in triplicate and expressed as the percentage of NP-treated cells over that of untreated control cells (100%).

Since Eu and Nd-doped samples boasted strong luminescence intensities in the NIR range (*vide supra*), they were considered promising for optical bioimaging applications. A preliminary analysis of the effects of undoped and Eu- and Nd-doped (at atomic percentages of 0.1, 1, and 5%, respectively) ZnS on cellular toxicity in human cells (A549 alveolar carcinoma cells) was carried out by means of the MTS assay (3-(4,5-dimethylthiazol-2-yl)-5-(3-carboxymethoxyphenyl)-2-(4-sulfophenyl)-2*H*-tetrazolium salt assay), which measures the reduction of tetrazolium salts to a water-soluble formazan product. The intracellular reduction of MTS is primarily attributable to mitochondrial dehydrogenases, and therefore, this conversion is used as a measure of cell viability. The results were compared with cells not treated with NPs (control) and showed that, in the tested concentration range (i.e., 0–400 μg/mL), undoped and Eu- and Nd-doped ZnS NPs did not affect cell viability (Figure 12a,b).

Since the MTS assay is based on the enzymatic activity of cellular dehydrogenases (mainly mitochondrial) that can be active also during the early stages of cell death, we further evaluated the cloning efficiency of NP-treated cells as a measure of cytotoxicity. The clonogenic assay is a very sensitive approach to assessing cell proliferation because it measures the cycling ability of viable and healthy cells and can therefore provide a better estimate of the cellular response of NP-treated cells. The doses for NP treatments analyzed here were selected based on dose–response curves assessed in our previous studies, which dealt with the same cells treated with different types of NPs.^18,70^ Our results showed that the capacity of cells to form colonies was not significantly affected by the incubation of undoped and doped zinc sulfide NPs (Figure 12c). The decrease of viability of cells incubated with the lowest dose (10 μg/mL) of undoped ZnS, ZnS:Eu 0.1 at. %, and ZnS:Nd 1 at. % is not significant and not accompanied by a further decrease of viability at higher doses (50 and 100 μg/mL), indicating the absence of cytotoxicity for these NP samples and, in general, for all the tested formulations of undoped and doped zinc sulfide NPs.

## Conclusions

In this work, the continuous-flow synthesis of very small (around 5 nm) crystalline and monodispersed undoped and doped zinc sulfide nanoparticles was successfully accomplished via microfluidic synthesis. Remarkably, small and stable nanoparticles were obtained without using any surfactant or ligand to hinder particle growth. This success was ascribed to the special features of the microfluidic approach. The dynamic nature of the system as well as the highly effective and rapid mixing of reactants combined with a quenching step allows the formation of primary non-aggregated particles with an average diameter of 5 nm.

First, the synthesis of Mn-doped ZnS nanoparticles was performed. Subsequently, having successfully obtained luminescent ZnS:Mn NPs, zinc sulfide was doped with lanthanide ions (i.e., Eu^3+^, Nd^3+^, Sm^3+^, and Yb^3+^). The NPs obtained displayed a crystalline cubic sphalerite ZnS phase, which was retained when introducing the dopant ions at different concentrations (0.1, 1, and 5 at. %), as evidenced by XRPD analyses. The dopants could not be detected on the surface of the samples (XPS), but their incorporation in the final products was confirmed by ICP-MS measurements. Eu L_3_- and Nd L_3_-XANES and EXAFS data on Eu- and Nd-doped samples showed that the dopant ions were not hosted within the sulfide matrix but rather formed separate moieties in which lanthanide ions are coordinated to oxygen. These separate phases were evidenced also by XRPD (Ln(OH)_3_ reflections in samples doped with the highest amounts of Ln^3+^) and confirmed the oxophilic nature of lanthanides. Nevertheless, Ln-doped samples featuring the desired luminescence properties were successfully obtained, displaying strong luminescence intensities in the near-infrared region, an important quality for *in vivo* optical bioimaging applications. In addition, a slight direct sensitization of lanthanide ions (Eu^3+^ and Nd^3+^) by the ZnS NPs was observed. This is a notable result as it was obtained through a simple room-temperature microfluidic approach. In view of employing the synthesized systems in the field of optical bioimaging, the effects of Eu- and Nd-doped samples on human cellular toxicity were evaluated *in vitro*. Overall, our results showed that ZnS NPs at the tested concentrations do not have cytotoxic effects on human *in vitro* cultured A549 cells, strengthening their appeal as possible contrast agents in bioimaging applications.

## Experimental Methods

### Chemicals

Zinc(II) nitrate hexahydrate (Zn(NO_3_)_2_·6H_2_O) was purchased from Fluka. Manganese(II) chloride tetrahydrate (MnCl_2_·4H_2_O), europium(III) chloride hexahydrate (EuCl_3_·6H_2_O), samarium(III) nitrate hexahydrate (Sm(NO_3_)_3_·6H_2_O), neodymium(III) nitrate hexahydrate (Nd(NO_3_)_3_·6H_2_O), ytterbium(III) chloride hexahydrate (YbCl_3_·6H_2_O), and sodium sulfide nonahydrate (Na_2_S·9H_2_O) were purchased from Sigma-Aldrich. All chemicals were used without further purification.

### Microfluidic Setup

**Figure 13:**
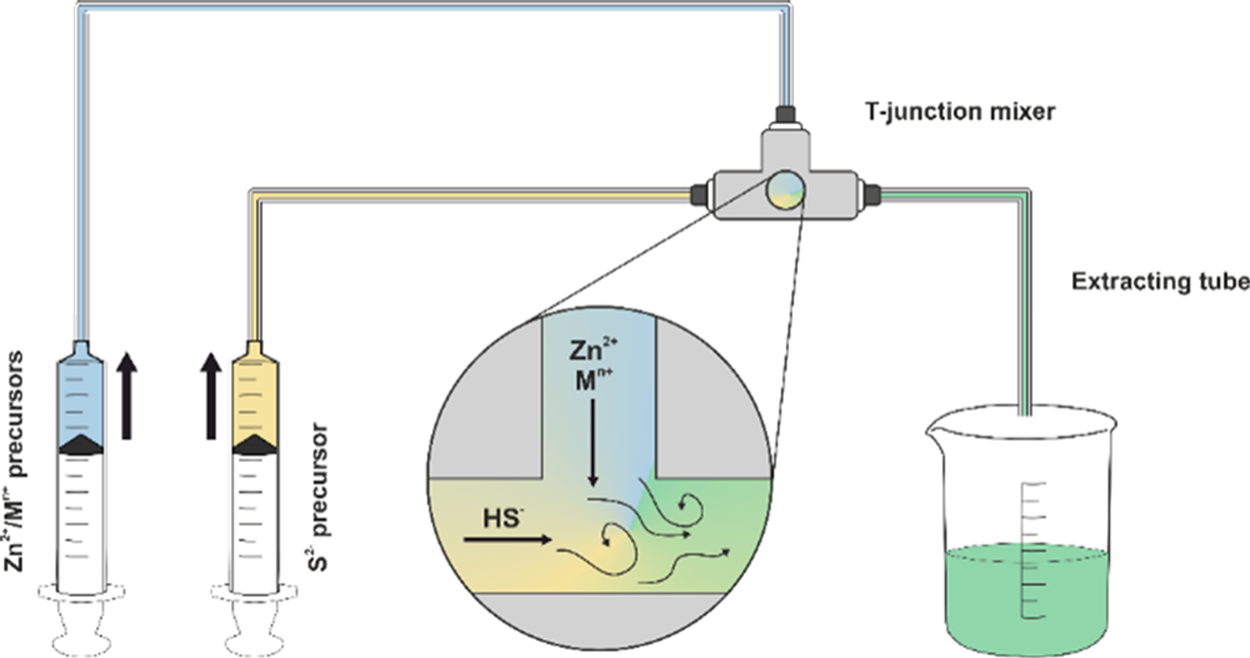
Schematic representation of the microfluidic setup used (HS^–^ is the predominant species at the native pH value of 0.2 M Na_2_S water solution).

The microfluidic setup was built using 0.63 mm diameter PTFE tubes and a T-shaped stainless-steel single mixer. A 0.1 M MilliQ water solution of zinc nitrate hexahydrate and dopant metal salt with the appropriate molar ratio (to yield the desired dopant atomic percentage) was employed as a metal precursor solution, while a 0.2 M sodium sulfide nonahydrate MilliQ water solution was employed as a sulfide precursor. Each solution was pumped separately into the reactor using a reciprocating syringe pump (Syrris Asia Syringe Pump) with a flow rate of 1.2 mL/min. The reaction system was kept at room temperature. A schematic representation of the setup is reported in Figure 13. The obtained slurry (ca. 150 mL) was collected in ca. 200 mL of cold MilliQ water, kept in an ice bath, and left to settle down overnight in order to easily remove most of the mother liquor. The slurry was then repeatedly centrifugated (10,000 rpm, 10 min) and washed with MilliQ water. The final powder was dried overnight in a vacuum desiccator at room temperature.

### X-Ray Powder Diffraction

The XRPD patterns of the doped ZnS nanostructures (as grinded powder) were collected with a Bruker D8 Advance diffractometer equipped with a Göbel mirror by using Cu Kα radiation. Diffractograms were recorded in the 10–80° 2θ range with a 0.1° 2θ scan step and a 10 s per step acquisition time. The angular accuracy was 0.0010°, and the angular resolution was better than 0.01°. All the experimental data were analyzed by using the Material Analysis Using Diffraction (MAUD) software package^71^ to deduce quantitative crystallographic and microstructural information by using the whole-powder pattern fitting (WPPF) method.^34^ The average crystallite size and the microstrain (root mean square of the variations in the lattice parameters) values were evaluated using an isotropic model. The quality of fitting was evaluated by means of the *R* indexes *R*_wp_ and *R*_exp_ and by the goodness of fit χ^2^. The refined lattice constant *a* was reported to two decimal places because of the low signal-to-noise ratio and broad peaks typical of nanocrystalline materials.^72^

### X-ray Photoelectron Spectroscopy

Powder samples (deposited on conductive tape) were investigated by XPS with a PerkinElmer φ 5600ci instrument using Al Kα radiation (1486.6 eV), operating at 350 W. The working pressure was less than 5 × 10^–8^ Pa. The calibration was based on the binding energy (BE) of the Au 4f_7/2_ line at 83.9 eV with respect to the Fermi level. The standard deviation for the BE values was 0.15 eV. Reported BEs were corrected for charging effects, and the BE value of 284.6 eV was assigned to the C 1s line of carbon.^44^ Survey scans were obtained in the 0–1350 eV range (pass energy of 187.5 eV, 1.0 eV/step, 25 ms/step). Detailed scans (29.35 eV pass energy, 0.1 eV/step, 50–150 ms/step) were recorded for O1s, C1s, Zn2p, ZnLMM, S2p, S2s, Mn2p, and Ln3d regions. The atomic composition, after a Shirley-type background subtraction,^73^ was evaluated using sensitivity factors supplied by PerkinElmer. Peak assignment was carried out according to literature data, and fitting of Zn2p and S2p regions was performed with KolXPD software.^74^

### Transmission Electron Microscopy

TEM micrographs were obtained with a FEI Tecnai G12 microscope operating at 100 kV, equipped with an OSIS Veleta camera. Samples were prepared by suspending the dried powders in MilliQ water through sonication and then depositing them on 300-mesh lacey carbon-coated copper grids. Particles were manually segmented and measured using the ImageJ package.^75^

### High-Resolution Transmission Electron Microscopy

HRTEM micrographs were obtained using an aberration (image)-corrected FEI Titan 80–300 TEM operating at 300 kV and equipped with a Gatan US1000 slow-scan CCD camera. Samples were prepared suspending the dried powders in a solution of 10^–3^ M of oleylamine in *n*-hexane (0.5 mg/mL) and subsequently ultrasonicating the dispersion at high power (280 W) using a tip sonicator.

### X-ray Absorption Spectroscopy

X-ray absorption experiments at the Eu and Nd L_3_ edges were performed at the XAFS beamline at Elettra Sincrotrone Trieste, operating at 2.4 GeV and 140 mA. For energy selection, a Si(311) double-crystal monochromator was used. For Eu-containing samples, the spectra were recorded in transmission mode using two ionization chambers, both upstream and downstream of the sample. Spectra were recorded in the range of 6782–7600 eV. In the case of measurements conducted at the Nd L_3_-edge (collection range 5913–6710 eV), the fluorescence collection mode with a silicon drift detector was employed. Samples were prepared by pressing the dry powders (using cellulose as binder) into homogeneous pellets with the aid of a hydraulic press.

The XAS output data were reduced and analyzed with the freeware Demeter package.^76^ The spectra were summed up, deglitched, background-subtracted, and calibrated to yield the EXAFS function χ(*k*) (up to *k* 12.5 Å^–1^ for Eu and 11 Å^–1^ for Nd). The edge positions were determined from the zero crossing of the second derivative spectra. The Artemis software was used to fit the EXAFS curves in the *R* space, which were Fourier-filtered over a 3–9 Å^–1^ range and weighted by a smooth Hanning function. The amplitude reduction factor (*S*_0_^2^) was calibrated against crystalline oxide references and determined to be 0.8 for both Eu and Nd. For fitting (on phase-corrected data), the threshold energy, *E*_0_, the interatomic distances, *R*, the number of atoms, *N*, in the first shell (either O or S) and Debye-Waller factors, σ^2^, were allowed to vary. For final fittings, a Ln(OH)_3_ model was used as a reference compound.

### Quantum Chemical Calculations

*Ab initio* quantum chemical calculations of Eu and Nd L_3_ edge XANES spectra were performed with the FEFF9.6 code based on the multiple scattering theory.^77^ The potentials of free atoms were calculated with a relativistic Dirac–Fock code. The scattering potentials were calculated self-consistently by overlapping the free atomic density in the muffin tin approximation within a cluster of approximately 30 atoms. The energy-dependent exchange Hedin–Lundquist potential was used for the fine structure and the atomic background. The full multiple scattering XANES spectra were calculated for an atomic cluster of approximately 40 atoms centered on the absorbing Ln atom.

### Absorption and Photoluminescence Spectroscopy

Diffuse reflectance spectrum of the undoped sample was measured with a Cary5000 spectrophotometer (300–800 nm). Photoluminescence spectra were carried out on powder samples with a Nanolog/Fluorolog-3-2iHR320 modular spectrofluorometer equipped with a xenon lamp (450 W, ozone-free) and an R928P (Hamamatsu) photomultiplier. The samples were prepared for the emission measurements simply by filling the appropriate sample holder adapted for the used spectrofluorometer. In this regard, it should be noted that the same amount of powders was present in the optical path for the photoluminescence measurements, assuring reproducibility of the luminescence measurements. Since the experiments were conducted in a front-face geometry at 22.5°, the artifacts in the excitation spectra due to the radiation penetration depth variations are negligible. Emission decay measurements were recorded using the xenon flash lamp as the excitation source. The average lifetimes were determined by the expression
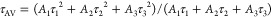
The near infrared emission was measured using a liquid nitrogen-cooled InGaS photodiode array (Horiba). The optical resolution for both the visible and near-infrared spectra is 1 nm. All the measurements were carried out at room temperature.

### ICP-MS Analysis

ICP-MS measurements were carried out with an Agilent Technologies 7700x ICP-MS (Agilent Technologies International Japan, Ltd., Tokyo, Japan). Operating conditions and data acquisition parameters were chosen according to a previous work.^78^

The internal standard mixture (Agilent, 5183-4681) containing Bi, Ge, In, Sc, Tb, Y, and ^6^Li (at 10 μg/mL each) in 3.5 wt % HNO_3_ was used. Multielement standard solutions (IV-ICPMS-71A INORGANIC VENTURES) for calibration were prepared by gravimetric serial dilution at nine different concentrations (from 10 to 1000 μg/L). The solvent used was 3.5 wt % HNO_3_ obtained from concentrated HNO_3_ and diluted with MilliQ water. Regression parameters of the calibration lines were obtained according to the Theil-Sen nonparametric regression technique.

#### Procedure of Digestion of Doped ZnS Nanoparticles

In order to correctly digest the samples, 5–10 mg of the analyte was dissolved in 5 g of 69 wt % HNO_3_ and heated in a water bath for 30 min at 100 °C. After cooling, solutions were diluted in order to obtain concentrations of the elements in the required calibration range and in a 3.5 wt % HNO_3_ solution.

### Cell Culture and Treatments with ZnS NPs

The human A549 cells (lung adenocarcinoma) were purchased from the American Type Culture Collection (ATCC n. CCL-185) and cultured in Ham’s F12-K nutrient mixture (Invitrogen Life Technologies, Carlsbad, CA, U.S.A.) supplemented with 10% heat-inactivated fetal bovine serum (FBS, Biochrom, Berlin, Germany), 38 units/mL streptomycin, and 100 units/mL penicillin G in T75 cm^2^ flasks (FALCON). Cells were kept at 37 °C in a humidified atmosphere of 95% air and 5% CO_2_ and maintained in an exponential and asynchronous phase of growth by repeated trypsinization and reseeding prior to reaching subconfluency. Cell treatments with NPs have been carried out as previously reported.^18,70^ In brief, the cells were seeded and maintained for 24 h in complete culture medium (10% FBS) before starting the NP treatment; then, the cells were incubated for 24 h in culture medium (3% FBS) in which the NP stock suspensions were freshly diluted. Control cells were subjected to the same treatments except for NP incubation. The NP stock solutions were diluted in ultrapure water (2 mg/mL), sonicated for 15 min using a homogenizer (Branson 3510 Ultrasonic Cleaner, Marshall Scientific, Hampton, NH, U.S.A.), and then sterilized by filtration with 0.22 μM immediately before use.

### Cell Viability

Cytotoxicity induced by ZnS NPs was evaluated by the MTS assay (CellTiter 96 Aqueous One Solution Cell Proliferation Assay, Promega, Madison, WI, U.S.A.) as previously described.^70^ In brief, 5 × 10^3^ cells were seeded in triplicate in 96-well plates (200 μL/well) in culture medium with FBS 10%. After 24 h, the culture medium was removed, and the cells were incubated with fresh culture medium containing 3% FBS and increasing concentrations of NPs (0–400 μg/mL). After 24 h, the medium containing NPs was removed, and the cells were incubated for 60–90 min in the dark with 20 μL of the MTS reagent diluted in 100 μL of serum-free medium. The absorbance of the formazan product was recorded at 490 nm with a microplate reader (Spectramax 190, Molecular Device). As the amount of 490 nm adsorbance is directly proportional to the number of living cells in culture, cell viability was determined by comparing the absorbance values of treated versus untreated control cells that were considered as 100%.

For clonogenic assays, A549 cells were seeded in 24-well plates and 24 h later were incubated with ZnS NPs as previously reported.^18^ NP-treated and untreated control cells were then trypsinized and plated in 60 × 15 mm dishes (500 cells/dish) in complete fresh medium. The cloning efficiency (CE) was calculated as the proportion of cells that formed colonies (greater than ≥50 cells) to the total number of cells plated, expressed in percentage. The CE values were then used to determine cell survival, expressed as the percentage of the CE of NP-treated cells over that of untreated control cells (100%).

## Supporting Information Available

XRPD patterns comparisons of undoped and doped samples, XRPD fittings and corresponding refined parameters, trend of average crystallite size and lattice parameter *a* as functions of the doping concentration, HRTEM and TEM micrographs and size distribution histograms, XPS fittings, surface atomic composition obtained from semi-quantitative analysis, XPS data of Sm- and Yb-doped samples, ICP-MS data of Sm- and Yb-doped ZnS samples, EXAFS fittings, UV–vis diffuse reflection spectrum of undoped ZnS, picture of undoped and Mn-doped samples under UV illumination, comparison between the absorption band of undoped ZnS and the excitation band of Mn-doped samples, and photoluminescence spectra of ZnS:Eu 5 at. % (PDF)

## Abbreviations

AP: Auger parameter
BE: binding energy
CN: coordination number
NPs: nanoparticles
NIR: near-infrared
EXAFS: extended X-ray absorption fine structure
DF-TEM: dark field transmission electron microscopy
HRTEM: high-resolution transmission electron microscopy
ICP-MS: inductively coupled plasma mass spectroscopy
MTS: (3-(4,5-dimethylthiazol-2-yl)-5-(3-carboxymethoxyphenyl)-2-(4-sulfophenyl)-2*H*-tetrazolium salt
RE: rare earth
TM: transition metal
XANES: X-ray absorption near-edge structure
XAS: X-ray absorption spectroscopy
XPS: X-ray photoelectron spectroscopy
XRPD: X-ray powder diffraction
WPPF: whole-powder pattern fitting

## References

[ref1] Thakur M.; Lentle B. C. Radiology Report of a Summit on Molecular Imaging. Radiology 2005, 236, 753–755. 10.1148/radiol.2363051160. 16118158

[ref2] Salmaso S.; Caliceti P. Stealth Properties to Improve Therapeutic Efficacy of Drug Nanocarriers. J. Drug Delivery 2013, 2013, 374252–374219. 10.1155/2013/374252. PMC3606770 23533769

[ref3] Kobayashi H.; Ogawa M.; Alford R.; Choyke P. L.; Urano Y. New Strategies for Fluorescent Probe Design in Medical Diagnostic Imaging. Chem. Rev. 2010, 2620–2640. 10.1021/cr900263j. 20000749 PMC3241938

[ref4] Villa I.; Vedda A.; Cantarelli I. X.; Pedroni M.; Piccinelli F.; Bettinelli M.; Speghini A.; Quintanilla M.; Vetrone F.; Rocha U.; Jacinto C.; Carrasco E.; Rodríguez F. S.; Juarranz Á.; del Rosal B.; Ortgies D. H.; Gonzalez P. H.; Solé J. G.; García D. J. 1.3 Μm Emitting SrF_2_:Nd^3+^ Nanoparticles for High Contrast in Vivo Imaging in the Second Biological Window. Nano Res. 2015, 8, 649–665. 10.1007/s12274-014-0549-1.

[ref5] Hawrysz D. J.; Sevick-Muraca E. M. Developments Toward Diagnostic Breast Cancer Imaging Using Near-Infrared Optical Measurements and Fluorescent Contrast Agents1. Neoplasia 2000, 2, 388–417. 10.1038/sj.neo.7900118. 11191107 PMC1507982

[ref6] Ntziachristos V.; Bremer C.; Weissleder R. Fluorescence imaging with near-infrared light: new technological advances that enable in vivo molecular imaging. Eur. Radiol. 2003, 13, 195–208. 10.1007/s00330-002-1524-x. 12541130

[ref7] Janssen Y. M.; Van Houten B.; Borm P. J.; Mossman B. T. Cell and Tissue Responses to Oxidative Damage. Lab. Invest. 1993, 69, 261–274. 8377469

[ref8] Selvan S. T.; Tan T. T. Y.; Yi D. K.; Jana N. R. Functional and Multifunctional Nanoparticles for Bioimaging and Biosensing. Langmuir 2010, 26, 11631–11641. 10.1021/la903512m. 19961213

[ref9] Fang X.; Zhai T.; Gautam U. K.; Li L.; Wu L.; Bando Y.; Golberg D. ZnS Nanostructures: From Synthesis to Applications. Prog. Mater. Sci. 2011, 56, 175–287. 10.1016/j.pmatsci.2010.10.001.

[ref10] Li H.; Li M.; Shih W. Y.; Lelkes P. I.; Shih W. H. Cytotoxicity Tests of Water Soluble ZnS and CdS Quantum Dots. J. Nanosci. Nanotechnol. 2011, 11, 3543–3551. 10.1166/jnn.2011.3803. 21776735

[ref11] Deng Z.; Tong L.; Flores M.; Lin S.; Cheng J.-X.; Yan H.; Liu Y. High-Quality Manganese-Doped Zinc Sulfide Quantum Rods with Tunable Dual-Color and Multiphoton Emissions. J. Am. Chem. Soc. 2011, 133, 5389–5396. 10.1021/ja110996c. 21405017 PMC3074539

[ref12] Yu Z.; Ma X.; Yu B.; Pan Y.; Liu Z. Synthesis and Characterization of ZnS:Mn/ZnS Core/Shell Nanoparticles for Tumor Targeting and Imaging in Vivo. J. Biomater. Appl. 2013, 28, 232–240. 10.1177/0885328212444642. 22532407

[ref13] Bhargava R. N.; Gallagher D.; Hong X.; Nurmikko A. Optical Properties of Manganese-Doped nanocrystals of ZnS. Phys. Rev. Lett. 1994, 72, 1–419. 10.1103/PhysRevLett.72.416. 10056425

[ref14] Yang H.; Santra S.; Holloway P. H. Syntheses and Applications of Mn-Doped II-VI Semiconductor Nanocrystals. J. Nanosci. Nanotechnol. 2005, 5, 1364–1375. 10.1166/jnn.2005.308. 16193951

[ref15] Sapra S.; Prakash A.; Ghangrekar A.; Periasamy N.; Sarma D. D. Emission Properties of Manganese-Doped ZnS Nanocrystals. J. Phys. Chem. B 2005, 109, 1663–1668. 10.1021/jp049976e. 16851141

[ref16] Hu H.; Zhang W. Synthesis and Properties of Transition Metals and Rare-Earth Metals Doped ZnS Nanoparticles. Opt. Mater. 2006, 28, 536–550. 10.1016/j.optmat.2005.03.015.

[ref17] Dolcet P.; Maurizio C.; Casarin M.; Pandolfo L.; Gialanella S.; Badocco D.; Pastore P.; Speghini A.; Gross S. An Effective Two-Emulsion Approach to the Synthesis of Doped ZnS Crystalline Nanostructures. Eur. J. Inorg. Chem. 2015, 2015, 706–714. 10.1002/ejic.201403004.

[ref18] De Fazio A. F.; Morgese G.; Mognato M.; Piotto C.; Pedron D.; Ischia G.; Causin V.; Rosenboom J.-G.; Benetti E. M.; Gross S. Robust and Biocompatible Functionalization of ZnS Nanoparticles by Catechol-Bearing Poly(2-Methyl-2-Oxazoline)S. Langmuir 2018, 34, 11534–11543. 10.1021/acs.langmuir.8b02287. 30170495

[ref19] Chan W. C. W.; Maxwell D. J.; Gao X.; Bailey R. E.; Han M.; Nie S. Luminescent Quantum Dots for Multiplexed Biological Detection and Imaging. Curr. Opin. Biotechnol. 2002, 13, 40–46. 10.1016/S0958-1669(02)00282-3. 11849956

[ref20] Polarz S. Shape Matters: Anisotropy of the Morphology of Inorganic Colloidal Particles - Synthesis and Function. Adv. Funct. Mater. 2011, 21, 3214–3230. 10.1002/adfm.201101205.

[ref21] Dengo N.; De Fazio A. F.; Weiss M.; Marschall R.; Dolcet P.; Fanetti M.; Gross S. Thermal Evolution of ZnS Nanostructures: Effect of Oxidation Phenomena on Structural Features and Photocatalytical Performances. Inorg. Chem. 2018, 57, 13104–13114. 10.1021/acs.inorgchem.8b01101. 30303381

[ref22] Whitesides G. M. The Origins and the Future of Microfluidics. Nature 2006, 442, 368–373. 10.1038/nature05058. 16871203

[ref23] Nightingale A. M.; DeMello J. C. Microfluidics: Segmented Flow Reactors for Nanocrystal Synthesis. Adv. Mater. 2013, 25, 1806 10.1002/adma.201370083. 23135743

[ref24] Lignos I.; Protesescu L.; Stavrakis S.; Piveteau L.; Speirs M. J.; Loi M. A.; Kovalenko M. V.; DeMello A. J. Facile Droplet-Based Microfluidic Synthesis of Monodisperse IV-VI Semiconductor Nanocrystals with Coupled in-Line NIR Fluorescence Detection. Chem. Mater. 2014, 26, 2975–2982. 10.1021/cm500774p.

[ref25] DeMello A. J. Control and Detection of Chemical Reactions in Microfluidic Systems. Nature 2006, 442, 394–402. 10.1038/nature05062. 16871207

[ref26] Tofighi G.; Gaur A.; Doronkin D. E.; Lichtenberg H.; Wang W.; Wang D.; Rinke G.; Ewinger A.; Dittmeyer R.; Grunwaldt J.-D. Microfluidic Synthesis of Ultrasmall AuPd Nanoparticles with a Homogeneously Mixed Alloy Structure in Fast Continuous Flow for Catalytic Applications. J. Phys. Chem. C 2018, 122, 1721–1731. 10.1021/acs.jpcc.7b11383.

[ref27] Hessel V.; Löwe H.; Schönfeld F. Micromixers-a Review on Passive and Active Mixing Principles. Chem. Eng. Sci. 2005, 60, 2479–2501. 10.1016/j.ces.2004.11.033.

[ref28] Elvira K. S.; i Solvas X. C.; Wootton R. C. R.; deMello A. J. The Past, Present and Potential for Microfluidic Reactor Technology in Chemical Synthesis. Nat. Chem. 2013, 5, 905–915. 10.1038/nchem.1753. 24153367

[ref29] DeMello J.; DeMello A. Focus Microscale Reactors: Nanoscale Products. Lab Chip 2004, 4, 11N–15N. 10.1039/B403638G. 15164461

[ref30] Schmidt W.; Bussian P.; Lindén M.; Amenitsch H.; Agren P.; Tiemann M.; Schüth F. Accessing Ultrashort Reaction Times in Particle Formation with SAXS Experiments: ZnS Precipitation on the Microsecond Time Scale. J. Am. Chem. Soc. 2010, 132, 6822–6826. 10.1021/ja101519z. 20426411

[ref31] Tiemann M.; Weiß Ö.; Hartikainen J.; Marlow F.; Lindén M. Early Stages of ZnS Nanoparticle Growth Studied by In-Situ Stopped-Flow UV Absorption Spectroscopy. ChemPhysChem 2005, 6, 2113–2119. 10.1002/cphc.200500163. 16208753

[ref32] Dengo N.; Faresin A.; Carofiglio T.; Maggini M.; Wu L.; Hofmann J. P.; Hensen E. J. M.; Dolcet P.; Gross S. Ligand-Free ZnS Nanoparticles: As Easy and Green as It Gets. Chem. Commun. 2020, 56, 8707–8710. 10.1039/D0CC01901A. 32613962

[ref33] Holleman A. F.; Wiberg E. Lehrbuch Der Anorganischen Chemie, 101th edit.; deGruyter & Co: Berlin, 1985.

[ref34] Scardi P.; Leoni M. Whole Powder Pattern Modelling. Acta Crystallogr. Sect. A: Found. Crystallogr. 2002, 58, 190–200. 10.1107/S0108767301021298. 11832590

[ref35] Dengo N. Ligand-Free Water-Based Approaches for the Synthesis of Metal Sulfides Nanostructures; University of Padova, 2019.

[ref36] Singh A.; Limaye M.; Singh S.; Lalla N. P.; Malek C. K.; Kulkarni S. A Facile and Fast Approach for the Synthesis of Doped Nanoparticles Using a Microfluidic Device. Nanotechnology 2008, 19, 245613 10.1088/0957-4484/19/24/245613. 21825825

[ref37] LaMer V. K.; Dinegar R. H. Theory, Production and Mechanism of Formation of Monodispersed Hydrosols. J. Am. Chem. Soc. 1950, 72, 4847–4854. 10.1021/ja01167a001.

[ref38] LaMer V. K. Nucleation in Phase Transitions. Ind. Eng. Chem. 1952, 44, 1270–1277. 10.1021/ie50510a027.

[ref39] Sugimoto T. Monodispersed Particles; Elsevier: Amsterdam, 2001.

[ref40] Sugimoto T. Spontaneous Nucleation of Monodisperse Silver Halide Particles from Homogeneous Gelatin Solution I: Silver Chloride. Colloids Surf., A 2000, 164, 183–203. 10.1016/S0927-7757(99)00366-0.

[ref41] Chu D. B. K.; Owen J. S.; Peters B. Nucleation and Growth Kinetics from LaMer Burst Data. J. Phys. Chem. A 2017, 121, 7511–7517. 10.1021/acs.jpca.7b08368. 28929758

[ref42] Greenwood N. N.; Earnshaw A. Chemistry of the Elements, 2nd Edition.; Butterworth-Heinemann: Oxford, 1998.

[ref43] Taylor C. E.; Garvey S. D.; Pemberton J. E. Carbon Contamination at Silver Surfaces: Surface Preparation Procedures Evaluated by Raman Spectroscopy and X-Ray Photoelectron Spectroscopy. Anal. Chem. 1996, 68, 2401–2408. 10.1021/ac950753h.

[ref44] Moulder J. M. F.; Stickle W. F.; Sobol P. E.; Bomben K. D. Handbook of X-Ray Photoelectron Spectroscopy – a Reference Book of Standard Spectra for Identification and Interpretation of XPS Data; Physical Electronics 1992.

[ref45] Naumkin A. V.; Kraut-Vass A.; Gaarenstroom S. W.; Powell C. J. NIST X-ray Photoelectron Spectroscopy database http://srdata.nist.gov/xps/.

[ref46] Jing L.; Kershaw S. V.; Li Y.; Huang X.; Li Y.; Rogach A. L.; Gao M. Aqueous Based Semiconductor Nanocrystals. Chem. Rev. 2016, 116, 10623–10730. 10.1021/acs.chemrev.6b00041. 27586892

[ref47] Gaarenstroom S. W.; Winograd N. Initial and Final State Effects in the ESCA Spectra of Cadmium and Silver Oxides. J. Chem. Phys. 1977, 67, 3500–3506. 10.1063/1.435347.

[ref48] Wagner C. D. Chemical Shifts of Auger Lines, and the Auger Parameter. Faraday Discuss. Chem. Soc. 1975, 60, 291–300. 10.1039/DC9756000291.

[ref49] Langer D. W.; Vesely C. J. Electronic Core Levels of Zinc Chalcogenides. Phys. Rev. B 1970, 2, 4885–4892. 10.1103/PhysRevB.2.4885.

[ref50] Dake L. S.; Baer D. R.; Zachara J. M. Auger Parameter Measurements of Zinc Compounds Relevant to Zinc Transport in the Environment. Surf. Interface Anal. 1989, 14, 71–75. 10.1002/sia.740140115.

[ref51] Vaughan D. J.; Becker U.; Wright K. Sulphide Mineral Surfaces: Theory and Experiment. Int. J. Miner. Process. 1997, 51, 1–14. 10.1016/S0301-7516(97)00035-5.

[ref52] Shannon R. D. Revised Effective Ionic Radii and Systematic Studies of Interatomic Distances in Halides and Chalcogenides. Acta Crystallogr., Sect. A: Found. Crystallogr. 1976, 32, 751–767. 10.1107/S0567739476001551.

[ref53] Cao J.; Yang J.; Zhang Y.; Yang L.; Wang Y.; Wei M.; Liu Y.; Gao M.; Liu X.; Xie Z. Optimized Doping Concentration of Manganese in Zinc Sulfide Nanoparticles for Yellow-Orange Light Emission. J. Alloys Compd. 2009, 486, 890–894. 10.1016/j.jallcom.2009.07.097.

[ref54] Kurnia F.; Hart J. N. Band-Gap Control of Zinc Sulfide: Towards an Efficient Visible-Light-Sensitive Photocatalyst. ChemPhysChem 2015, 16, 2397–2402. 10.1002/cphc.201500264. 26080007

[ref55] Chen L.; Zhang J.; Luo Y.; Lu S.; Wang X. Effect of Zn^2+^ and Mn^2+^ Introduction on the Luminescent Properties of Colloidal ZnS:Mn^2+^ Nanoparticles. Appl. Phys. Lett. 2004, 84, 112–114. 10.1063/1.1638901.

[ref56] Bol A. A.; Meijerink A. Long-Lived Mn^2+^ Emission in Nanocrystalline ZnS:Mn^2+^. Phys. Rev. B 1998, 58, R15997–R16000. 10.1103/PhysRevB.58.R15997.

[ref57] Hoshina T.; Kawai H. Luminescence Excitation Spectra and Their Exciton Structures of ZnS Phosphors. Mn, (Cu, Al), (Ag, Al) and (Au, Al) Doped Phosphors. Jpn. J. Appl. Phys. 1980, 19, 267–277. 10.1143/jjap.19.267.

[ref58] Komada S.; Kobayashi T.; Arao Y.; Tsuchiya K.; Mori Y. Optical Properties of Manganese-Doped Zinc Sulfide Nanoparticles Classified by Size Using Poor Solvent. Adv. Powder Technol. 2012, 23, 872–877. 10.1016/j.apt.2012.09.007.

[ref59] Bol A. A.; Meijerink A. Luminescence Quantum Efficiency of Nanocrystalline ZnS:Mn^2+^. 1. Surface Passivation and Mn^2+^ Concentration. J. Phys. Chem. B 2001, 105, 10197–10202. 10.1021/jp0107560.

[ref60] Sotelo-Gonzalez E.; Roces L.; Garcia-Granda S.; Fernandez-Arguelles M. T.; Costa-Fernandez J. M.; Sanz-Medel A. Influence of Mn^2+^ Concentration on Mn^2+^–Doped ZnS Quantum Dot Synthesis: Evaluation of the Structural and Photoluminescent Properties. Nanoscale 2013, 5, 9156–9161. 10.1039/C3NR02422A. 23921811

[ref61] Zheng J.; Yuan X.; Ikezawa M.; Jing P.; Liu X.; Zheng Z.; Kong X.; Zhao J.; Masumoto Y. Efficient Photoluminescence of Mn^2+^ Ions in MnS/ZnS Core/Shell Quantum Dots. J. Phys. Chem. C 2009, 113, 16969–16974. 10.1021/jp906390y.

[ref62] Qian L.; Bera D.; Holloway P. Photoluminescence from ZnS/CdS:Mn/ZnS Quantum Well Quantum Dots. Appl. Phys. Lett. 2008, 92, 093103 10.1063/1.2889446.

[ref63] Park W.; Jones T. C.; Tong W.; Schön S.; Chaichimansour M.; Wagner B. K.; Summers C. J. Luminescence Decay Kinetics in Homogeneously and Delta-Doped ZnS:Mn. J. Appl. Phys. 1998, 84, 6852–6858. 10.1063/1.368980.

[ref64] Gumlich H.-E. Electro- and Photoluminescence Properties of Mn^2+^ in ZnS and ZnCdS. J. Lumin. 1981, 23, 73–99. 10.1016/0022-2313(81)90191-5.

[ref65] Beltran-Huarac J.; Wang J.; Tanaka H.; Jadwisienczak W. M.; Weiner B. R.; Morell G. Stability of the Mn Photoluminescence in Bifunctional ZnS:0.05Mn Nanoparticles. J. Appl. Phys. 2013, 114, 053106 10.1063/1.4817371.

[ref66] Suhling K.; Hirvonen L. M.; Levitt J. A.; Chung P.-H.; Tregidgo C.; Le Marois A.; Rusakov D. A.; Zheng K.; Ameer-Beg S.; Poland S.; Coelho S.; Henderson R.; Krstajic N. Fluorescence Lifetime Imaging (FLIM): Basic Concepts and Some Recent Developments. Med. Photonics 2015, 27, 3–40. 10.1016/j.medpho.2014.12.001.

[ref67] Hazra C.; Sarkar S.; Meesaragandla B.; Mahalingam V. Eu^3+^ Ions as an Optical Probe to Follow the Growth of Colloidal ZnO Nanostructures. Dalton Trans. 2013, 42, 11981–11986. 10.1039/C3DT51506K. 23846348

[ref68] Mukherjee P.; Shade C. M.; Yingling A. M.; Lamont D. N.; Waldeck D. H.; Petoud S. Lanthanide Sensitization in II–VI Semiconductor Materials: A Case Study with Terbium(III) and Europium(III) in Zinc Sulfide Nanoparticles. J. Phys. Chem. A 2011, 115, 4031–4041. 10.1021/jp109786w. 21090795 PMC3061249

[ref69] Bünzli J.-C. G.; Eliseeva S. V. Basics of Lanthanide Photophysics. In Lanthanide Luminescence: Photophysical, Analytical and Biological Aspects; Hänninen P., Härmä H., Eds.; Springer-Verlag Berlin Heidelberg, 2010; pp. 1–45.

[ref70] Fede C.; Selvestrel F.; Compagnin C.; Mognato M.; Mancin F.; Reddi E.; Celotti L. The Toxicity Outcome of Silica Nanoparticles (Ludox®) Is Influenced by Testing Techniques and Treatment Modalities. Anal. Bioanal. Chem. 2012, 404, 1789–1802. 10.1007/s00216-012-6246-6. 23053168 PMC3462312

[ref71] Lutterotti L. Total Pattern Fitting for the Combined Size–Strain–Stress–Texture Determination in Thin Film Diffraction. Nucl. Instruments Methods Phys. Res. Sect. B Beam Interact. Mater. Atoms 2010, 268, 334–340. 10.1016/j.nimb.2009.09.053.

[ref72] Holder C. F.; Schaak R. E. Tutorial on Powder X-Ray Diffraction for Characterizing Nanoscale Materials. ACS Nano 2019, 13, 7359–7365. 10.1021/acsnano.9b05157. 31336433

[ref73] Shirley D. A. High-Resolution X-Ray Photoemission Spectrum of the Valence Bands of Gold. Phys. Rev. B 1972, 5, 4709–4714. 10.1103/PhysRevB.5.4709.

[ref74] Libra J. KolXPD: Spectroscopy Data Measurement and Processing https://www.kolibrik.net/kolxpd/.

[ref75] Schneider C. A.; Rasband W. S.; Eliceiri K. W. NIH Image to ImageJ: 25 Years of Image Analysis. Nat. Methods 2012, 9, 671–675. 10.1038/nmeth.2089. 22930834 PMC5554542

[ref76] Ravel B.; Newville M. ATHENA, ARTEMIS, HEPHAESTUS: Data Analysis for X-Ray Absorption Spectroscopy Using IFEFFIT. J. Synchrotron Radiat. 2005, 12, 537–541. 10.1107/S0909049505012719. 15968136

[ref77] Rehr J. J.; Kas J. J.; Vila F. D.; Prange M. P.; Jorissen K. Parameter-Free Calculations of X-Ray Spectra with FEFF9. Phys. Chem. Chem. Phys. 2010, 12, 5503–5513. 10.1039/B926434E. 20445945

[ref78] Badocco D.; Lavagnini I.; Mondin A.; Favaro G.; Pastore P. Definition of the Limit of Quantification in the Presence of Instrumental and Non-Instrumental Errors. Comparison among Various Definitions Applied to the Calibration of Zinc by Inductively Coupled Plasma–Mass Spectrometry. Spectrochim. Acta Part B 2015, 114, 81–86. 10.1016/j.sab.2015.10.004.

