# Supplementary material for: Microfluidic Crystallization of Surfactant-Free Doped Zinc Sulfide Nanoparticles for Optical Bioimaging Applications
Source: ACS Appl Mater Interfaces. 2020 Sep 2;12(39):44074–87. doi: 10.1021/acsami.0c13150 (PMC8011799; doi:10.1021/acsami.0c13150)
Supplement: Supplementary file 1 — am0c13150_si_001.pdf [file am0c13150_si_001.pdf]

SUPPORTING INFORMATION

MICROFLUIDIC CRYSTALLIZATION OF SURFACTANT-FREE DOPED ZINC SULFIDE NANOPARTICLES FOR OPTICAL BIOIMAGING APPLICATIONS

Francesca Tajoli,[a,b] Nicola Dengo,[a,b] Maddalena Mognato,[c] Paolo Dolcet,[d] Giacomo Lucchini,[e] Andrea Faresin,[a] Jan-Dierk Grunwaldt,[d] Xiaohui Huang,[f] Denis Badocco,[a] Michele Maggini,[a,b] Christian Kübel,[f,g] Adolfo Speghini,[e]* Tommaso Carofiglio,[a,b]* and Silvia Gross[a,b]*

[a]Dipartimento di Scienze Chimiche, Università degli Studi di Padova – Via Marzolo 1, 35131 Padova, Italy

[b]INSTM, UdR di Padova – Via Marzolo 1, 35131 Padova, Italy

[c]Dipartimento di Biologia, Università degli Studi di Padova – Via Bassi 58B, 35131 Padova, Italy

[d]Karlsruher Institut für Technologie (KIT), Institut für Technische Chemie und Polymerchemie (ITCP) – Engesserstr. 20, 76131 Karlsruhe, Germany

[e]NRG, Dipartimento of Biotecnologie, Università di Verona and INSTM, RU Verona – Strada Le Grazie 15, 37314 Verona, Italy

[f]Karlsruher Institut für Technologie (KIT), Institut für Nanotechnologie (INT) – Hermann-von-Helmholtz-Platz 1, 76344 Eggenstein-Leopoldshafen, Germany

[g]Department of Materials & Earth Sciences, Technical University Darmstadt, Alarich-Weiss-Str. 2, 64287 Darmstadt, Germany

In Supporting Information, the following analyses are reported:

- X-ray Powder Diffraction: fittings of XRPD patterns of undoped and doped ZnS samples (Figures S1, S2, S4, S6, S8, S10) and corresponding refined parameters (Table S1-S6), average crystallite size and lattice constant *a* as functions of dopant concentrations (Figures S3, S5, S7, S9, S11), XRPD patterns of Nd-, Sm- and Yb-doped ZnS samples compared with undoped one (Figure S12 and S13);
- Transmission Electron Microscopy: HR-TEM micrographs of undoped and Eu-doped ZnS samples (Figure S14 and S17) and TEM micrographs of doped ZnS samples and size distribution histograms (Figure S15, S16, S18-S20);
- X-ray Photoelectroon Spectroscopy: fittings of XPS peaks of undoped and 5 at% doped ZnS samples (dopants: Mn, Eu, Sm, Yb) (Figure S21-S25), surface atomic composition % of undoped and 5 at% doped samples (semi-quantitative analysis data) (Table S7), XPS data (binding energies and Auger parameters) of the Sm- and Yb-doped samples (Table S8);
- ICP-MS Analysis: ICP-MS data of the Sm- and Yb-doped samples (Table S9);
- X-ray Absorption spectroscopy: fitted Fourier transformed EXAFS data of Eu- and Nd-doped samples (Figures S26 and S27);
- Absorption and photoluminescence spectroscopy: UV-vis diffuse reflection spectrum of undoped ZnS (Figure S28), picture of undoped and Mn-doped samples under UV illumination (Figure S29), comparison between absorption band of undoped ZnS and excitation band of Mn-doped samples (Figure S30), photoluminescence spectra of ZnS:Eu 5 at% (Figure S31).

XRPD Diffraction.

Undoped ZnS

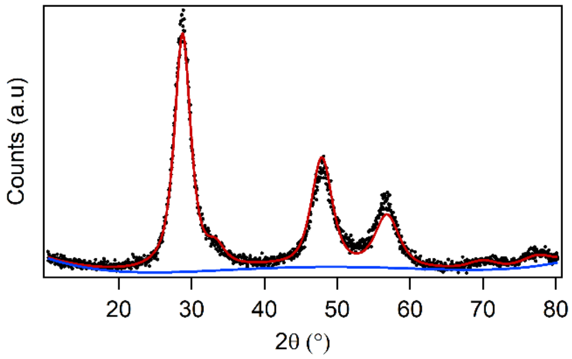

Figure S1. Fitting (red line) of diffractogram of undoped ZnS. Blue line: background.

Table S1. Refined parameters obtained from fitting of diffractogram of undoped ZnS.

Sample	Crystallite size (nm)	R.m.s. microstrain (%)	*a* (Å)	R_{exp} (%)	R_{wp} (%)	χ^2
Undoped ZnS	4.6 ± 0.1	1.5 ± 0.1	5.37 ± 0.01	9.58	12.76	1.77

Mn-doped ZnS

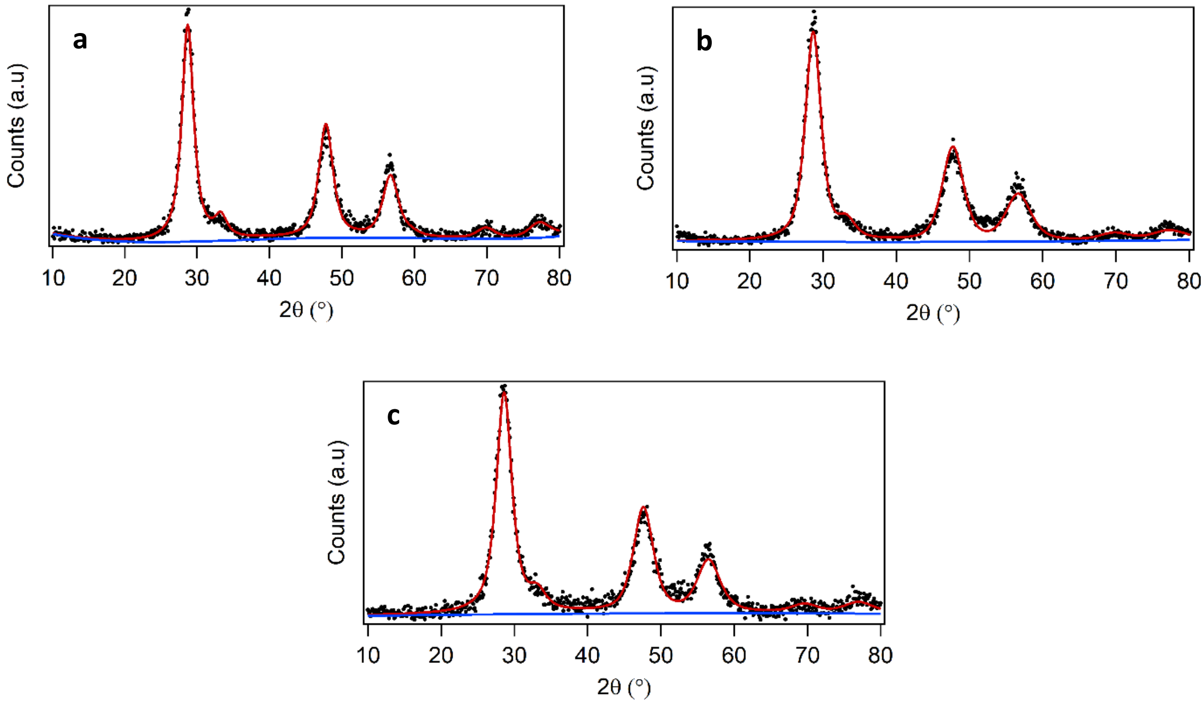

Figure S2. Fitting (red line) of diffractograms of Mn-doped ZnS samples. Doping percentages: 0.1 at% (a), 1 at% (b), 5 at% (c). Blue line: background.

Table S2. Refined parameters obtained from fitting of diffractogram of Mn-doped ZnS.

Sample	Crystallite size (nm)	R.m.s. microstrain (%)	*a* (Å)	R_{exp} (%)	R_{wp} (%)	χ^2
ZnS:Mn 0.1 at%	5.9 ± 0.1	0.8 ± 0.1	5.38 ± 0.01	12.13	14.14	1.36
ZnS Mn 1 at%	5.4 ± 0.2	1.8 ± 0.1	5.39 ± 0.01	10.05	13.91	1.91
ZnS:Mn 5 at%	4.8 ± 0.1	1.4 ± 0.1	5.40 ± 0.01	10.10	12.37	1.50

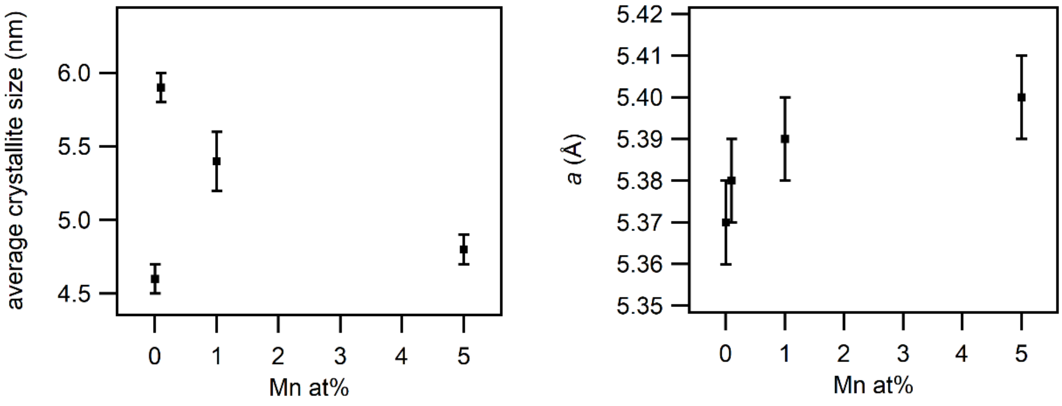

Figure S3. Average crystallite size (left) and lattice constant *a* (right) obtained from fitting of diffractograms of Mn-doped ZnS as a function of Mn atomic percentage.

Eu-doped ZnS

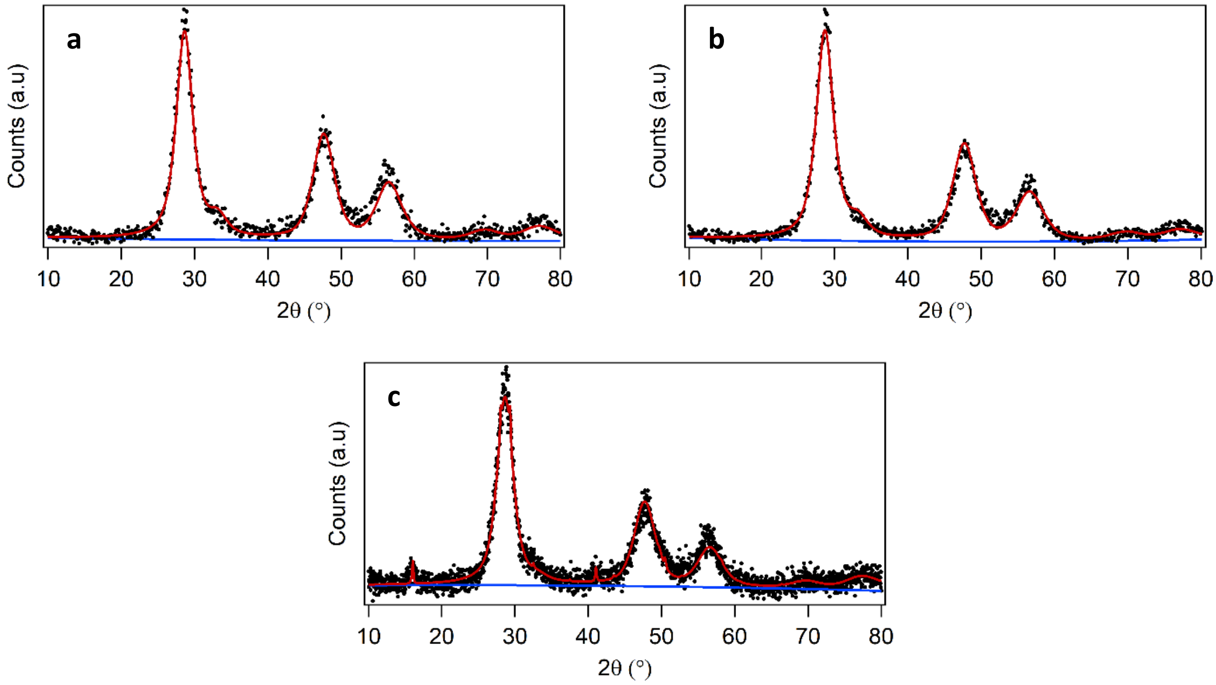

Figure S4. Fitting (red line) of diffractograms of Eu-doped ZnS samples. Doping percentages: 0.1 at% (a), 1 at% (b), 5 at% (c). Blue line: background.

Table S3. Refined parameters obtained from fitting of diffractogram of Eu-doped ZnS.

Sample	Crystallite size (nm)	R.m.s. microstrain (%)	*a* (Å)	R_{exp} (%)	R_{wp} (%)	χ^2	$Eu(OH)_3$ wt%
ZnS:Eu 0.1 at%	4.9 ± 0.2	1.8 ± 0.1	5.41 ± 0.01	12.29	14.63	1.42	-
ZnS:Eu 1 at%	4.6 ± 0.1	1.8 ± 0.1	5.39 ± 0.01	9.06	11.72	1.68	-
ZnS:Eu 5 at%	5.1 ± 0.2	1.7 ± 0.1	5.39 ± 0.01	10.50	11.32	1.16	1.8 ± 0.2

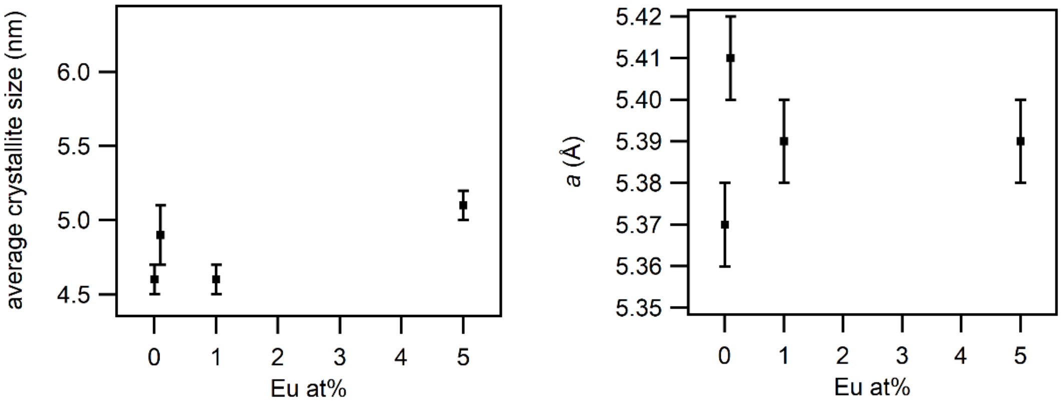

Figure S5. Average crystallite size (left) and lattice constant *a* (right) obtained from fitting of diffractograms of Eu-doped ZnS as a function of Eu atomic percentage.

Nd-doped ZnS

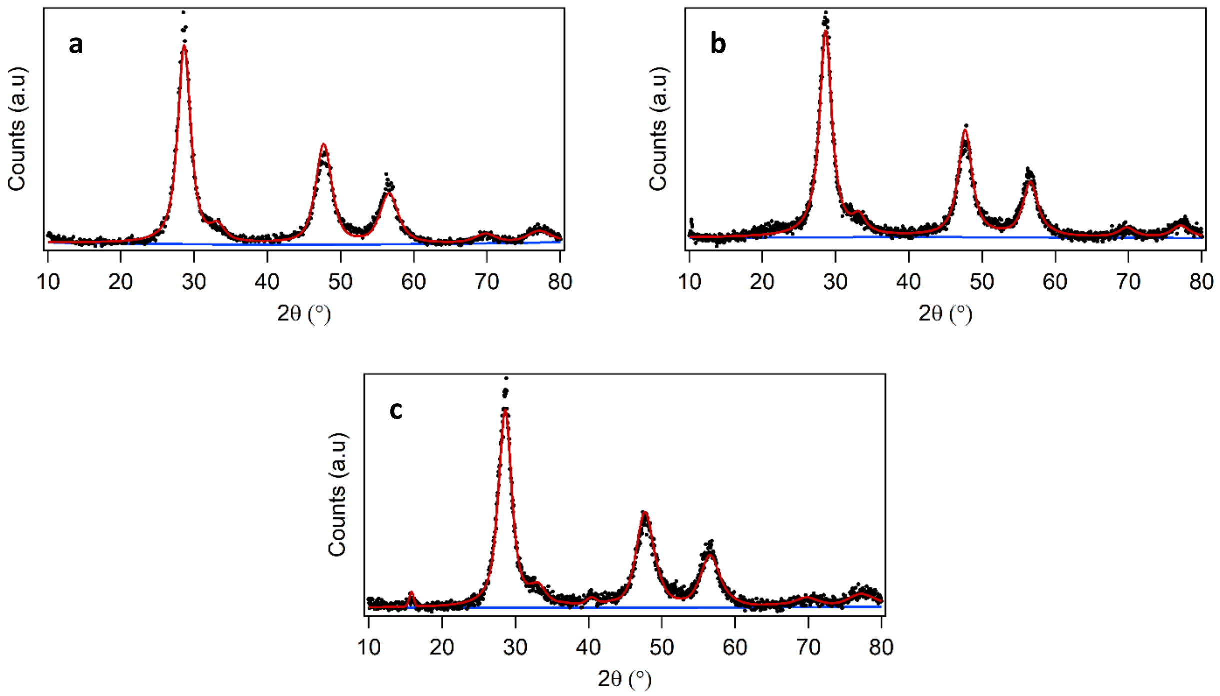

Figure S6. Fitting (red line) of diffractograms of Nd-doped ZnS samples. Doping percentages: 0.1 at% (a), 1 at% (b), 5 at% (c). Blue line: background.

Table S4. Refined parameters obtained from fitting of diffractogram of Nd-doped ZnS

Sample	Crystallite size (nm)	R.m.s. microstrain (%)	*a* (Å)	R_{exp} (%)	R_{wp} (%)	χ^2	$Nd(OH)_3$ wt%
ZnS:Nd 0.1 at%	5.7 ± 0.1	1.1 ± 0.1	5.39 ± 0.01	10.06	14.21	2.00	-
ZnS:Nd 1 at%	5.3 ± 0.1	0.5 ± 0.1	5.39 ± 0.01	9.75	12.76	1.71	-
ZnS:Nd 5 at%	5.5 ± 0.1	1.2 ± 0.1	5.40 ± 0.01	9.74	12.52	1.65	3.6 ± 0.2

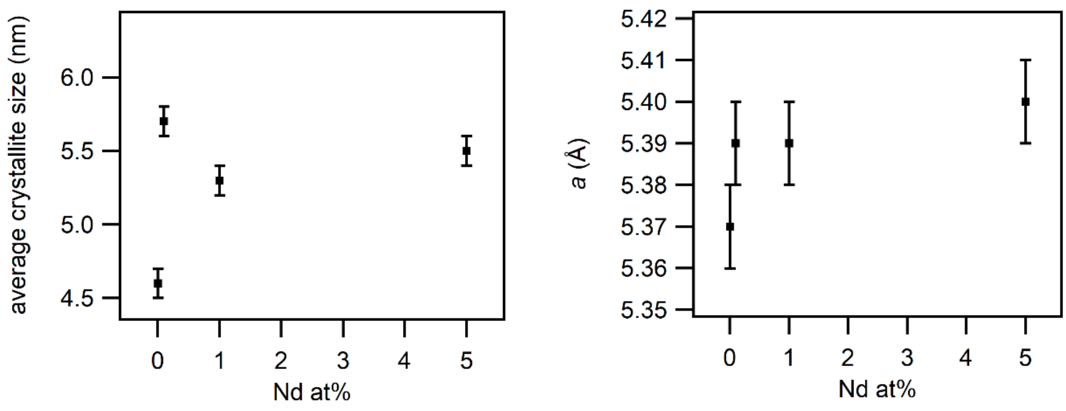

Figure S7. Average crystallite size (left) and lattice constant *a* (right) obtained from fitting of diffractograms of Nd-doped ZnS as a function of Nd atomic percentage.

Sm-doped ZnS

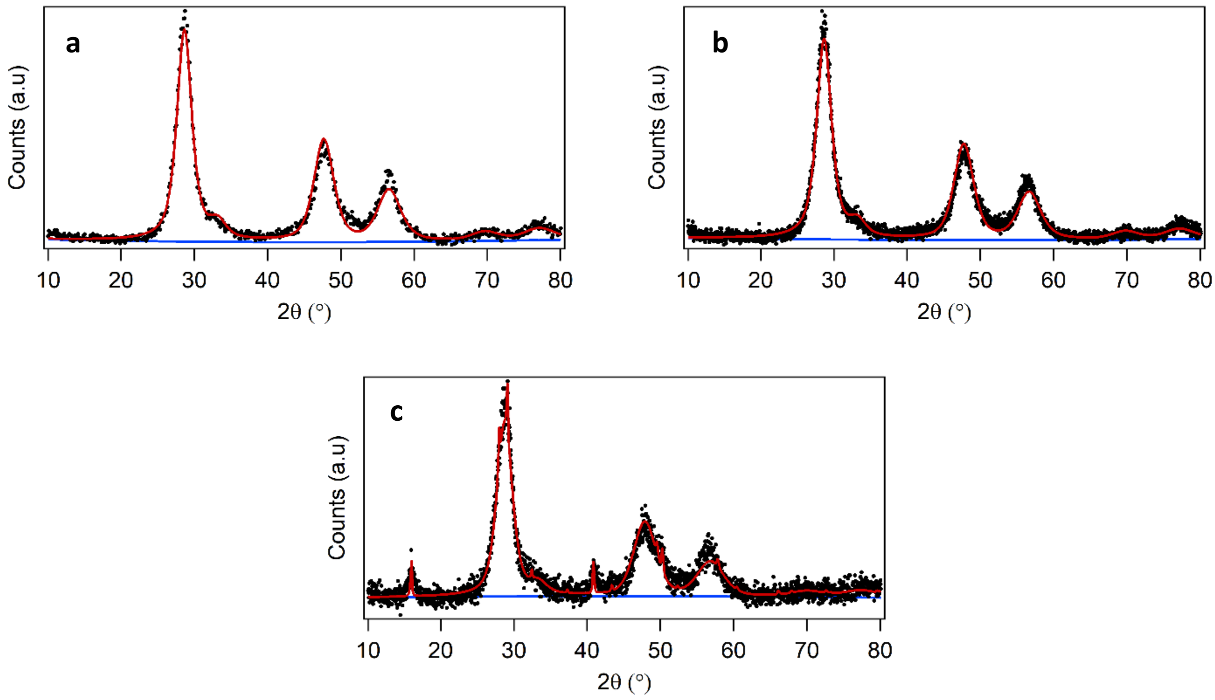

Figure S8. Fitting (red line) of diffractograms of Sm-doped ZnS samples Doping percentages: 0.1 at% (a), 1 at% (b), 5 at% (c). Blue line: background.

Table S5. Refined parameters obtained from fitting of diffractogram of Sm-doped ZnS.

Sample	Crystallite size (nm)	R.m.s. microstrain (%)	a (Å)	Rexp (%)	Rwp (%)	χ2	Sm(OH)3 wt%
ZnS:Sm 0.1 at%	5.4 ± 0.2	1.6 ± 0.1	5.40 ± 0.01	10.14	14.89	2.20	-
ZnS:Sm 1 at%	5.4 ± 0.1	1.6 ± 0.1	5.39 ± 0.01	11.73	15.22	1.68	-
ZnS:Sm 5 at%	6.2 ± 0.2	2.1 ± 0.1	5.37 ± 0.01	12.17	14.38	1.40	3.2 ± 0.2

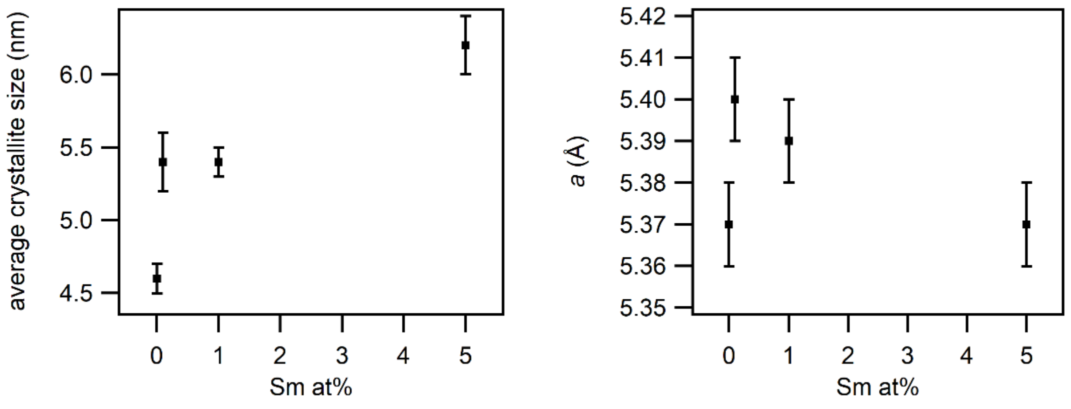

Figure S9. Average crystallite size (left) and lattice constant *a* (right) obtained from fitting of diffractograms of Sm-doped ZnS as a function of Sm atomic percentage.

Yb-doped ZnS

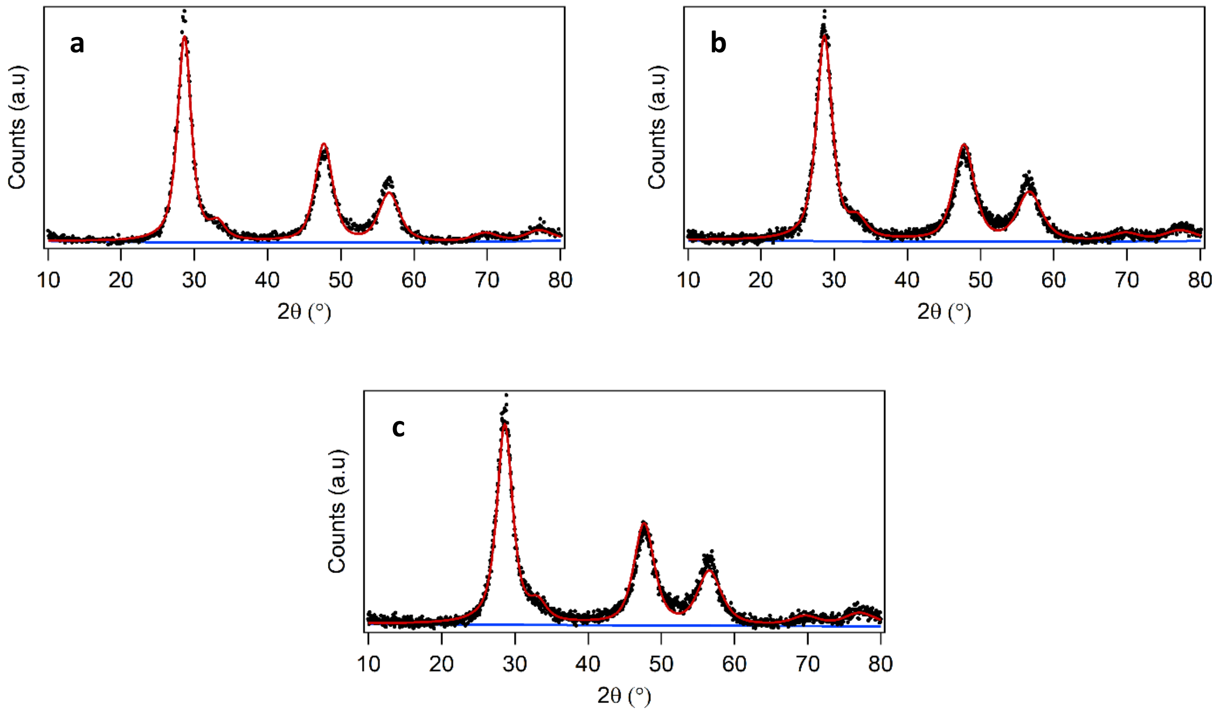

Figure S10. Fitting (red line) of diffractograms of Yb-doped ZnS samples. Doping percentages: 0.1 at% (a), 1 at% (b), 5 at% (c). Blue line: background.

Table S6. Refined parameters obtained from fitting of diffractogram of Yb-doped ZnS.

Sample	Crystallite size (nm)	R.m.s. microstrain (%)	*a* (Å)	R_{exp} (%)	R_{wp} (%)	χ^2
ZnS:Yb 0.1 at%	5.7 ± 0.2	1.4 ± 0.1	5.40 ± 0.01	9.41	14.34	2.32
ZnS:Yb 1 at%	5.0 ± 0.1	1.6 ± 0.1	5.39 ± 0.01	9.56	13.19	1.90
ZnS:Yb 5 at%	4.9 ± 0.1	1.5 ± 0.1	5.40 ± 0.01	10.06	14.01	1.94

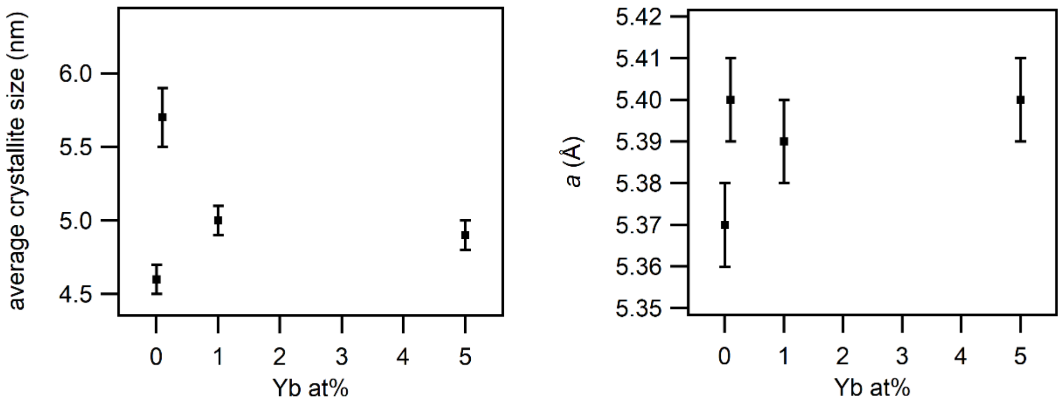

Figure S11. Average crystallite size (left) and lattice constant *a* (right) obtained from fitting of diffractograms of Yb-doped ZnS as a function of Yb atomic percentage.

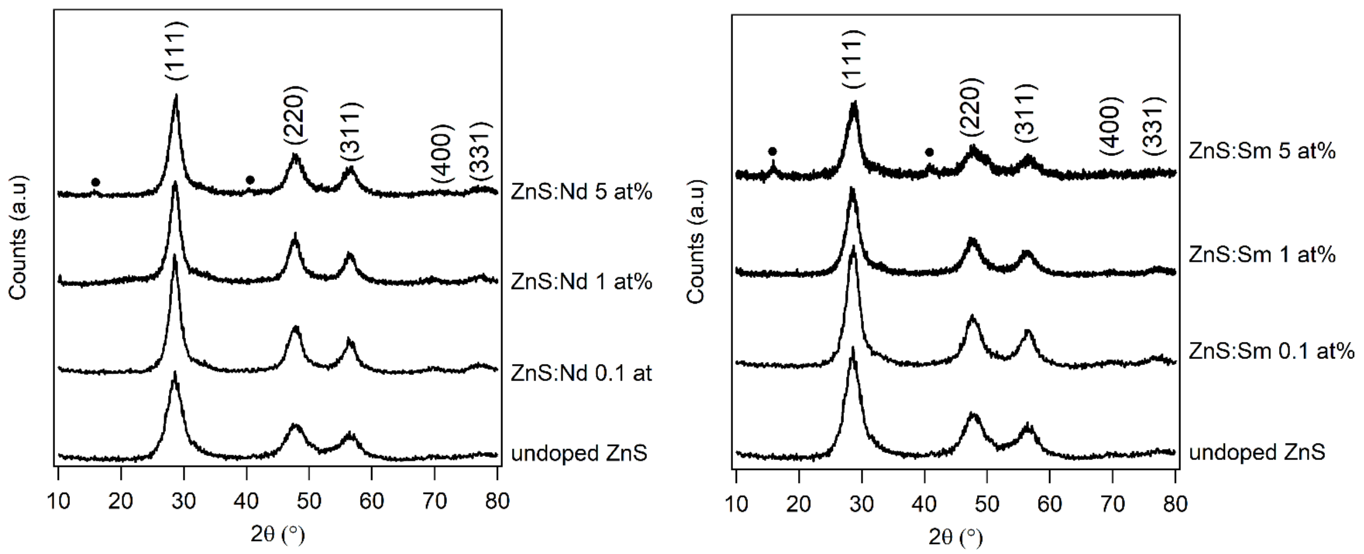

Figure S12. Comparison of diffractograms of undoped and Nd-doped (left), Sm-doped (right) at different atomic percentages. $Ln(OH)_3$ reflections are highlighted by a •.

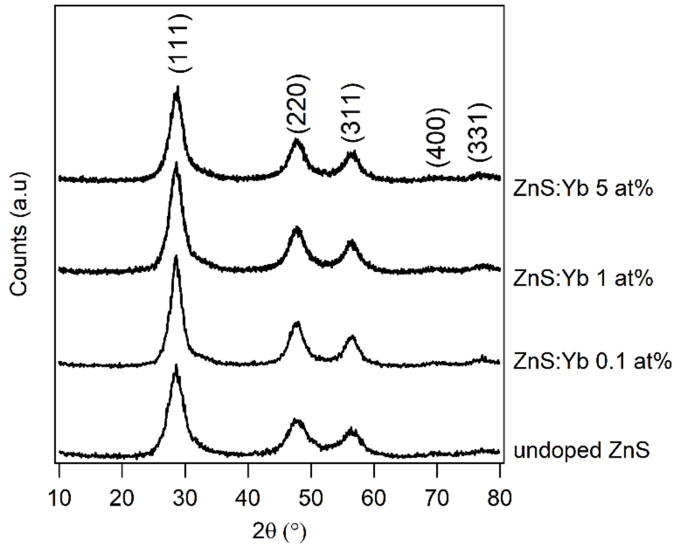

Figure S13. Comparison of diffractograms of undoped and Yb-doped ZnS samples at different atomic percentages.

Transmission Electron Microscopy and High-Resolution Transmission Electron Microscopy.

Undoped ZnS

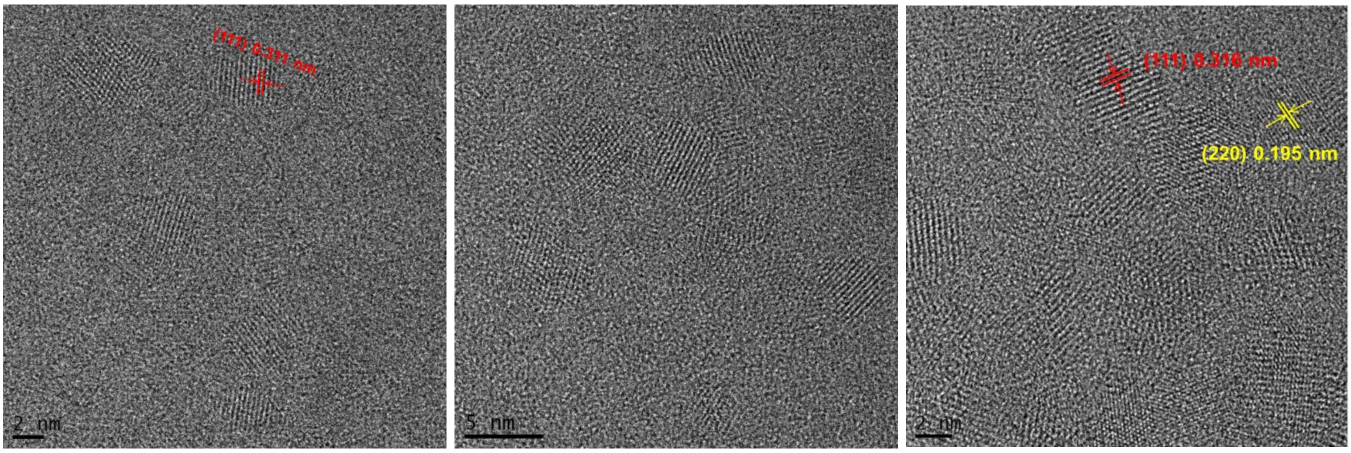

Figure S14. HRTEM micrographs of undoped zinc sulfide nanoparticles.

ZnS:Mn 5 at%

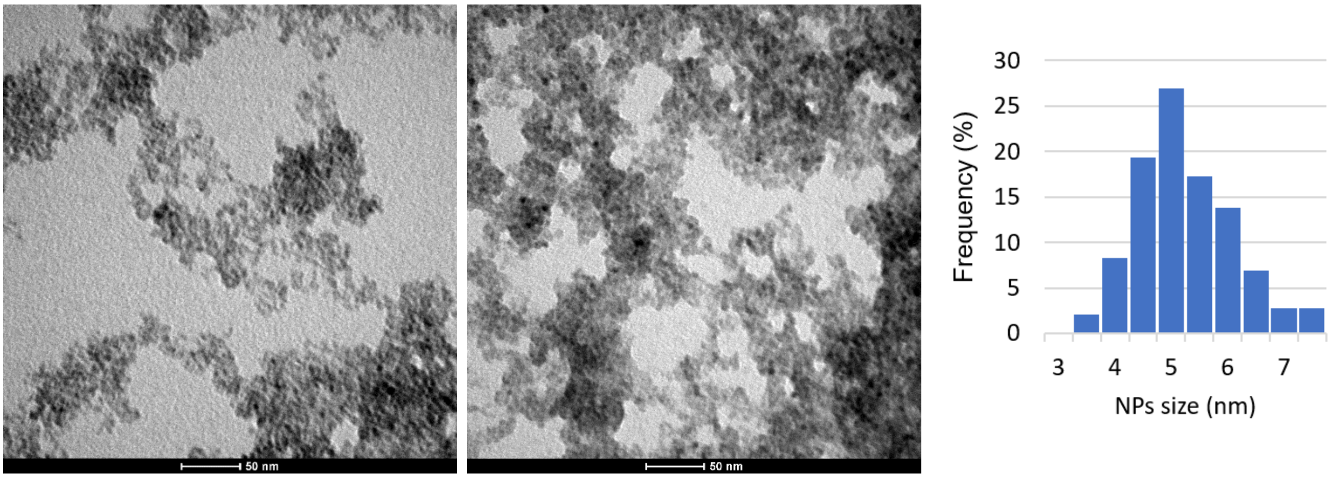

Figure S15. TEM micrographs of 5 at% Mn-doped zinc sulfide nanoparticles (left and center) and size distribution histogram (right).

ZnS:Eu 5 at%

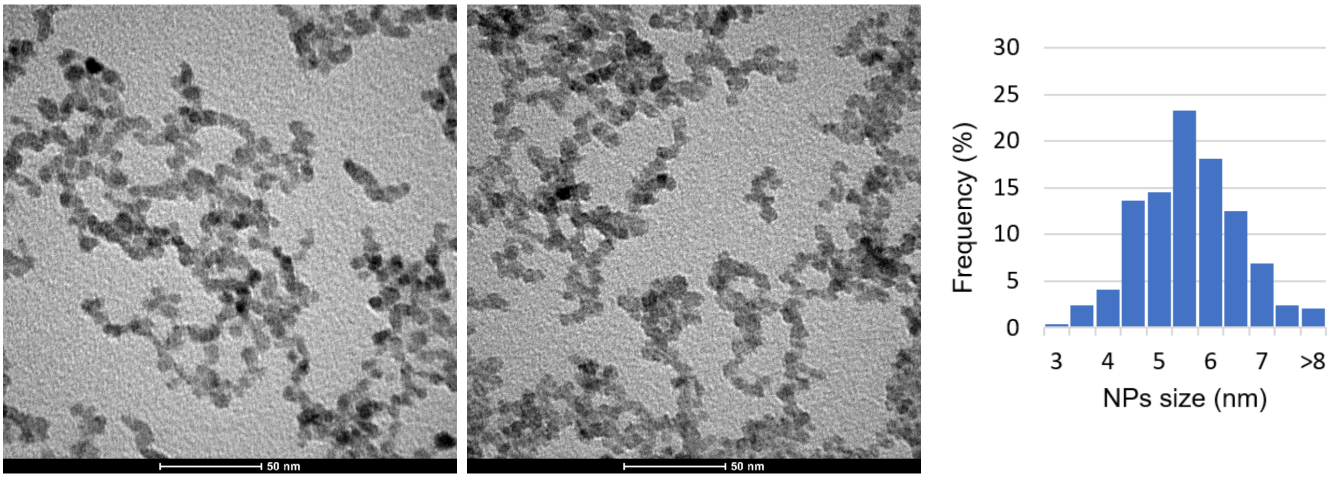

Figure S16. TEM micrographs of 5 at% Eu-doped zinc sulfide nanoparticles (left and center) and size distribution histogram (right).

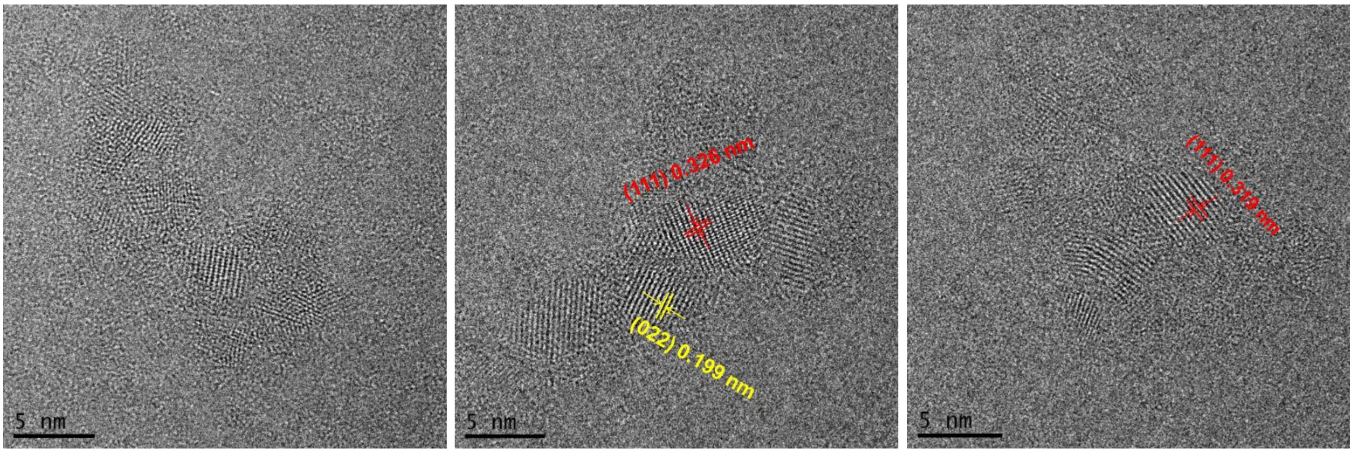

Figure S17. HRTEM micrographs of 5 at% Eu-doped zinc sulfide nanoparticles.

ZnS:Nd 5 at%

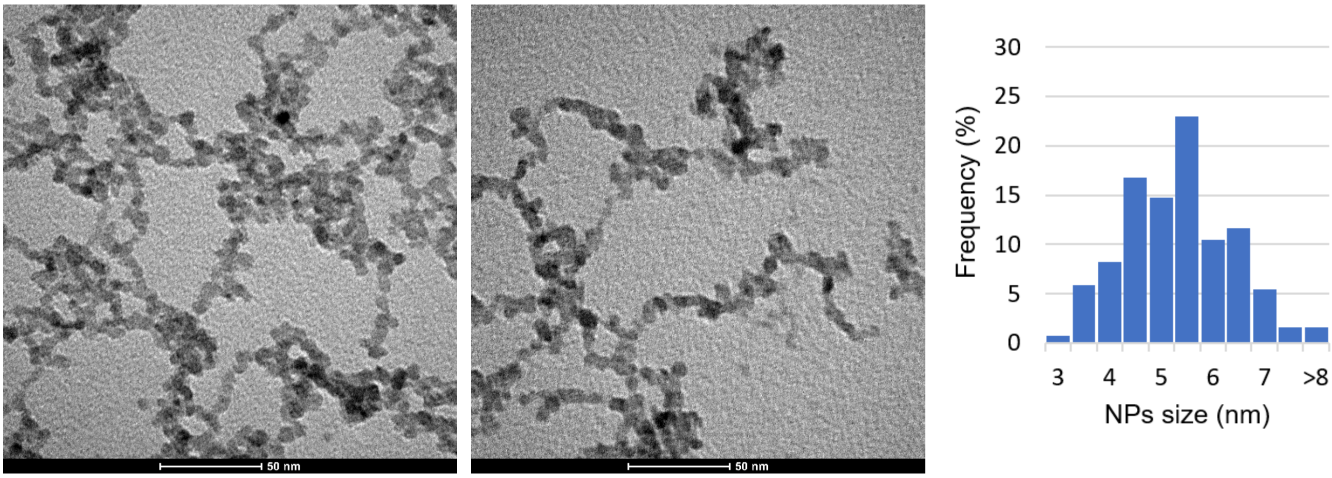

Figure S18. TEM micrographs of 5 at% Nd-doped zinc sulfide nanoparticles (left and center) and size distribution histogram (right).

ZnS:Sm 5 at%

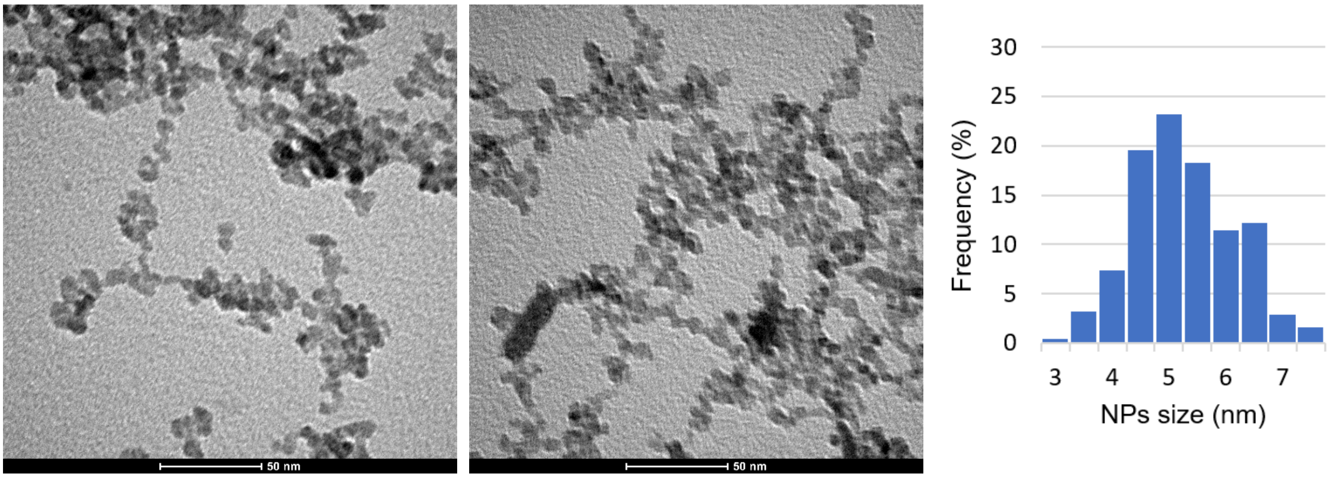

Figure S19. TEM micrographs of 5 at% Sm-doped zinc sulfide nanoparticles (left and center) and size distribution histogram (right).

ZnS:Yb 5 at%

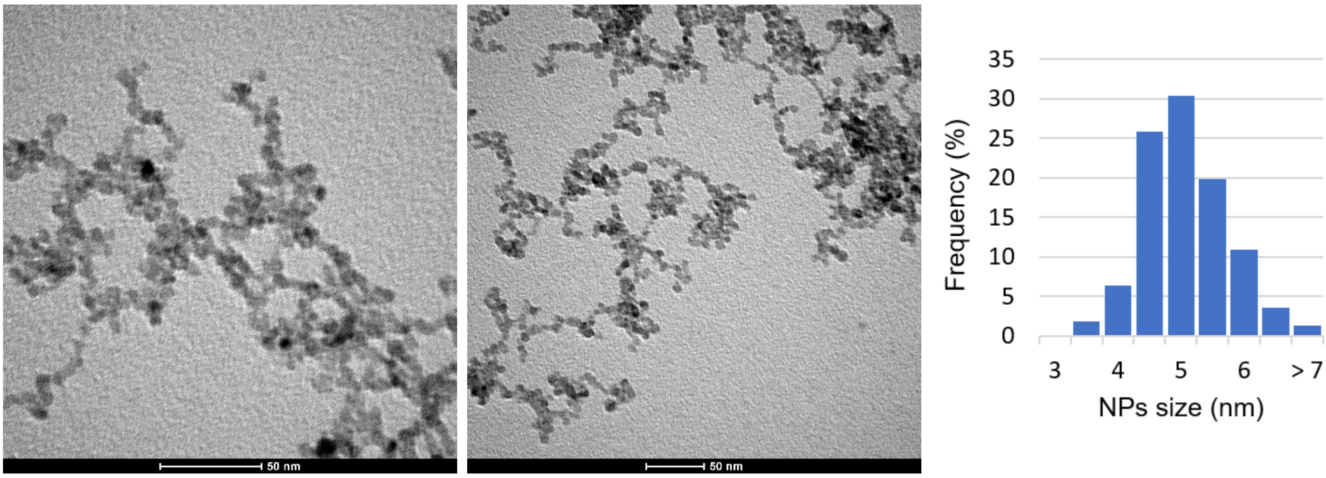

Figure S20. TEM micrographs of 5 at% Yb-doped zinc sulfide nanoparticles (left and center) and size distribution histogram (right).

X-ray Photoelectron Spectroscopy.

Undoped ZnS

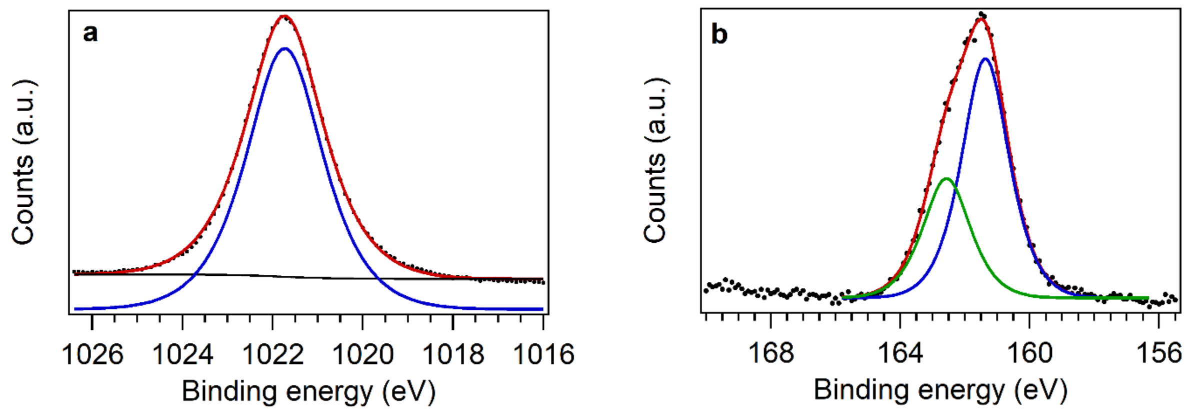

Figure S21. Fittings (red lines) of $Zn2p_{3/2}$ XPS peak (a) (blue line: photoemission peak) and S2p XPS peak (b) (blue line: $S2p_{3/2}$ component; green line: $S2p_{1/2}$ component, separated for clarity) of undoped ZnS. BE values are corrected for charge effects.

ZnS:Mn 5 at%

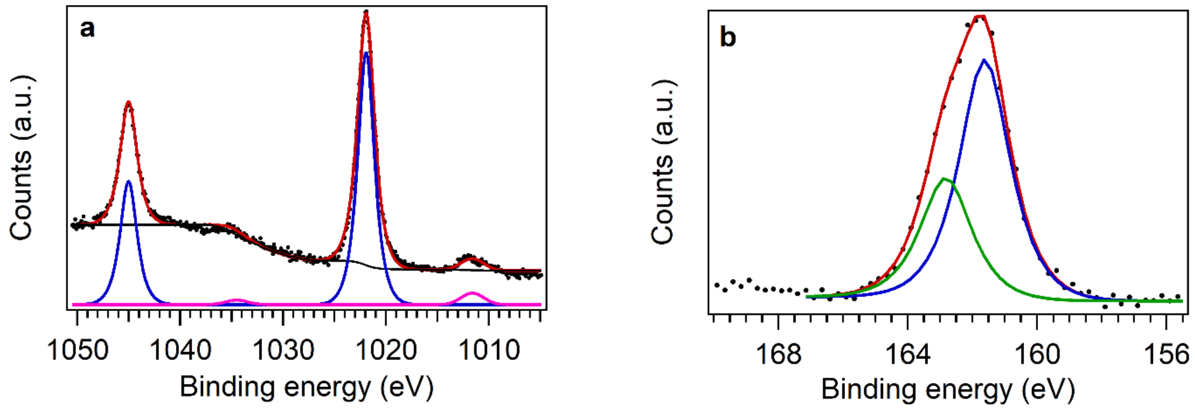

Figure S22. Fittings (red lines) of Zn2p XPS peaks (a) (blue line: photoemission peaks; pink line: shake up satellite peaks) and of S2p XPS peak (b) (blue line: $S2p_{3/2}$ component; green line: $S2p_{1/2}$ component, separated for clarity) of ZnS:Mn 5 at%. BE values are corrected for charge effects.

ZnS:Eu 5 at%

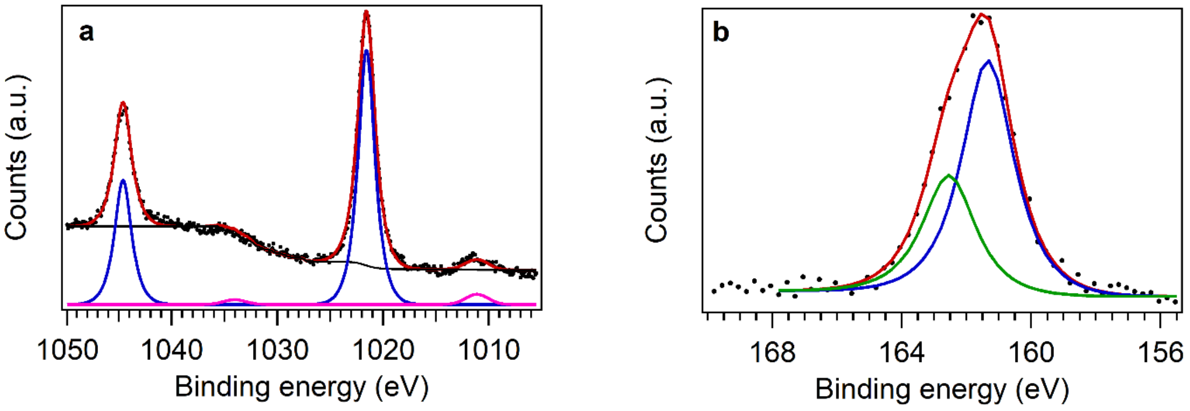

Figure S23. Fittings (red lines) of Zn2p XPS peaks (a) (blue line: photoemission peaks; pink line: shake up satellite peaks) and of S2p XPS peak (b) (blue line: $S2p_{3/2}$ component; green line: $S2p_{1/2}$ component, separated for clarity) of ZnS:Eu 5 at%. BE values are corrected for charge effects.

ZnS:Sm 5 at%

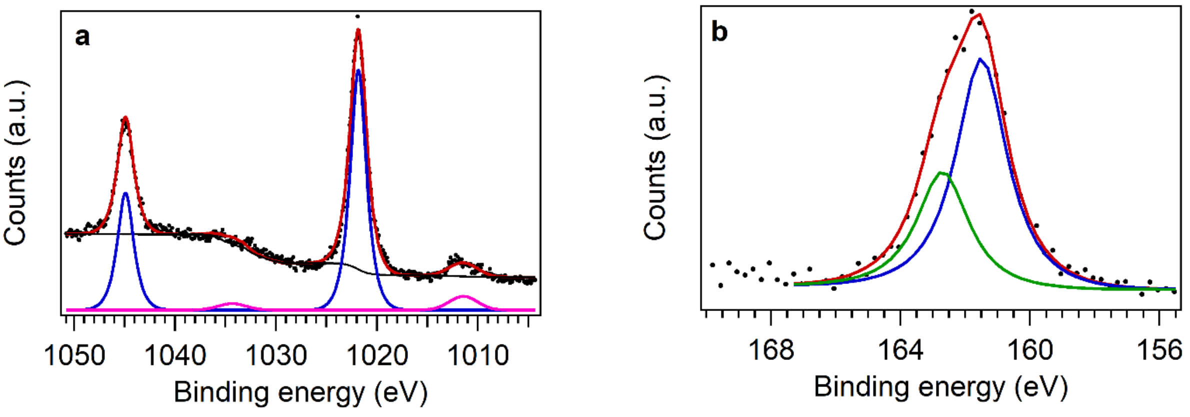

Figure S24. Fittings (red lines) of Zn2p XPS peaks (a) (blue line: photoemission peaks; pink line: shake up satellite peaks) and of S2p XPS peak (b) (blue line: $S2p_{3/2}$ component; green line: $S2p_{1/2}$ component, separated for clarity) of ZnS:Sm 5 at%. BE values are corrected for charge effects.

ZnS:Yb 5 at%

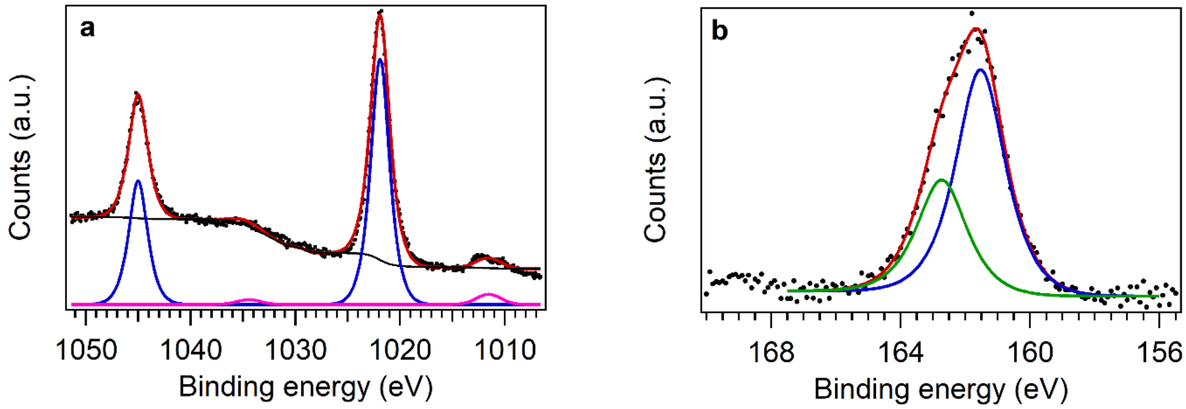

Figure S25. Fitting (red lines) of Zn2p XPS peaks (a) (blue line: photoemission peaks; pink line: shake up satellite peaks) and of S2p XPS peak (b) (blue line: $S2p_{3/2}$ component; green line: $S2p_{1/2}$ component, separated for clarity) of ZnS:Yb 5 at%. BE values are corrected for charge effects

Table S7 – Surface atomic composition (%) obtained from semi-quantitative analysis of ZnS2p3/2 and S2p XPS peaks.

Sample	Zn	S
Undoped ZnS	1	0.8
ZnS:Mn 5 at%	1	0.8
ZnS:Eu 5 at%	1	0.7
ZnS:Nd 5 at%	1	0.8
ZnS:Sm 5 at%	1	0.7
ZnS:Yb 5 at%	1	0.5

Table S8 – Binding energy (BE) and Auger parameter (AP) values.

Sample	BE $Zn2p_{3/2}$ (eV)	AP (eV)	BE S2p (eV)
ZnS:Sm 5 at%	1021.6	2011.4	161.4
ZnS:Yb 5 at%	1021.9	2011.5	161.4

ICP-MS Analysis.

Table S9. ICP-MS measurements (error: ± 5%).

	Atomic percentage $\frac{[\mathbf{M}]}{[\mathbf{Zn}] + [\mathbf{M}]} \cdot \mathbf{100}$	
	**Nominal**	**Experimental**
ZnS:Sm	0.1	0.09
	1	0.90
	5	5.44
ZnS:Yb	0.1	0.08
	1	0.48
	5	2.65

X-ray Absorption Spectroscopy.

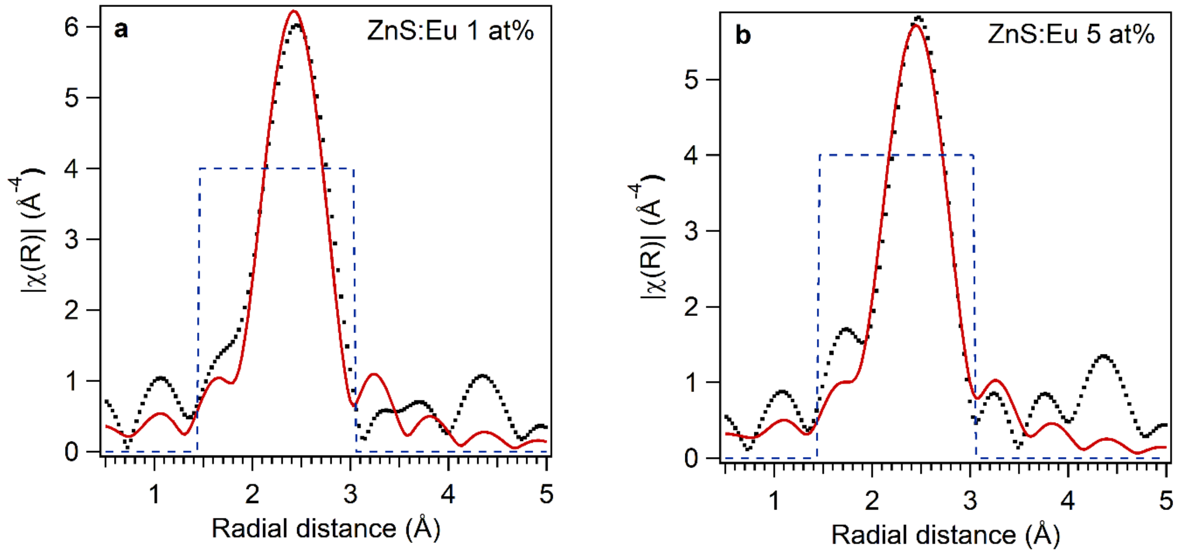

Figure S26. Fitted Fourier transformed EXAFS data of ZnS:Eu 1 at% (a) and ZnS:Eu 5 at% (b). Blue line: window for fitting range. Data are corrected for scattering phase-shift.

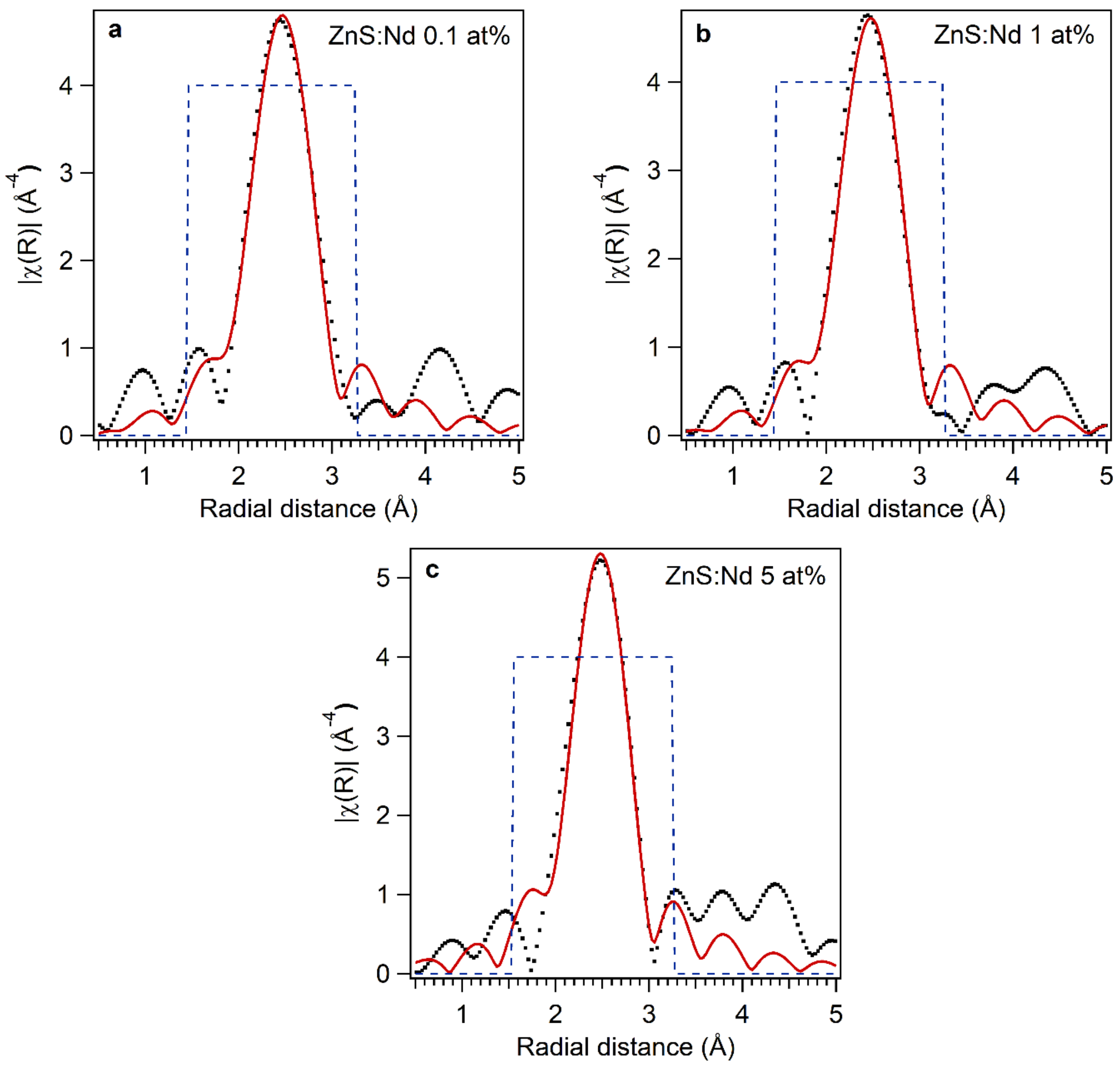

Figure S27. Fitted Fourier transformed EXAFS data of ZnS:Nd 0.1 at% (a), ZnS:Nd 1 at% (b) and ZnS:Nd 5 at% (c). Blue line: window for fitting range. Data are corrected for scattering phase-shift.

Absorption and photoluminescence spectroscopy.

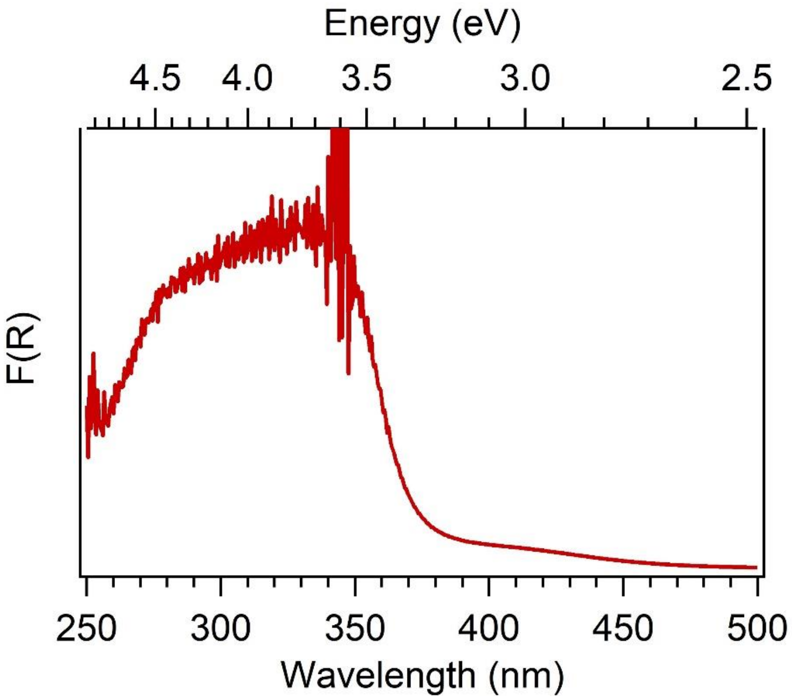

Figure S28. Diffuse reflectance spectrum of undoped ZnS sample.

The reflectance data in the 3.55-3.65 eV (350-340 nm) range are affected by some noise due to the change of the lamp of the spectrophotometer during the measurement and therefore in this part of the spectrum the signal shows relevant oscillations, as it can be noted in Figure S26. Nonetheless, an increasing behaviour of the reflectance is clearly observed from the visible region up to 330-340 nm, followed by a decrease of the reflectance on increasing the photon energy.

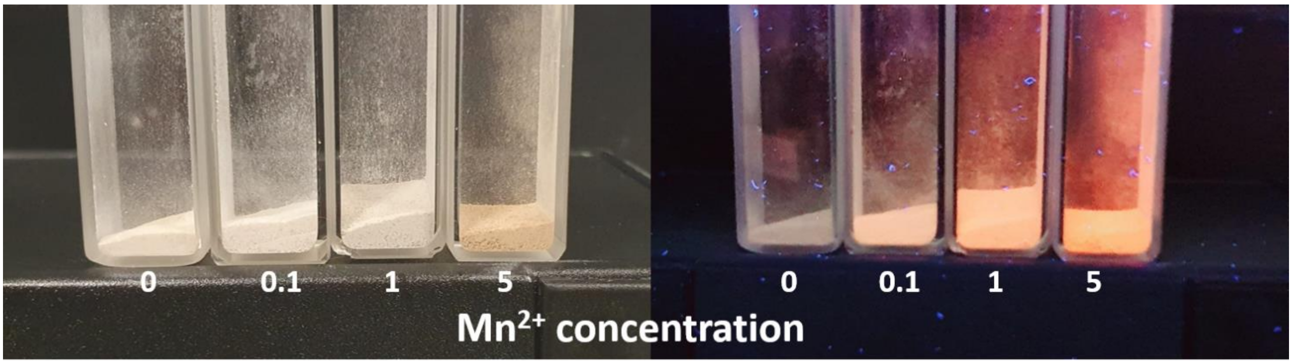

Figure S29. Picture of undoped and Mn-doped ZnS samples at different atomic concentrations, before (left) and during (right) UV illumination.

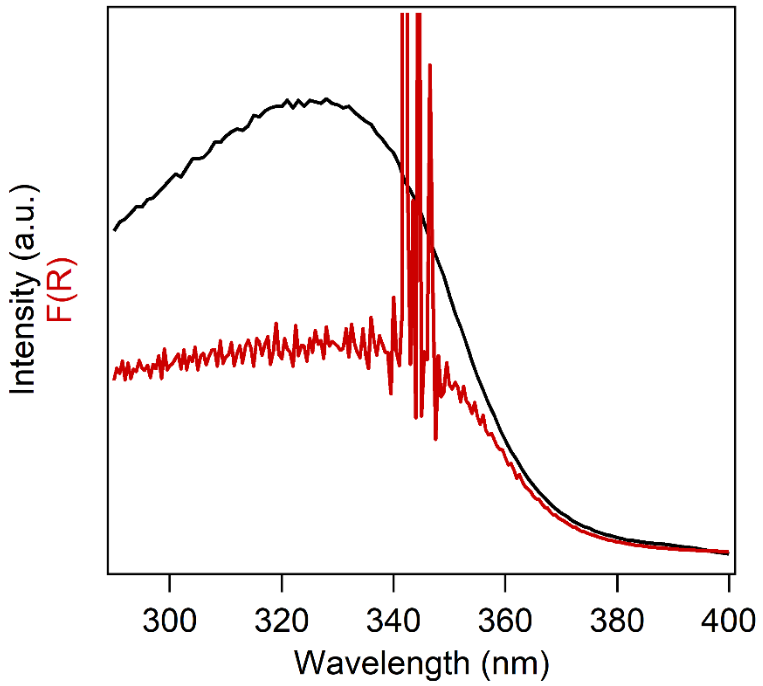

Figure S30. Comparison between absorption band of undoped ZnS (red line) and excitation band of 0.1 at% Mn-doped sample (black line, λ_{em}=597 nm).

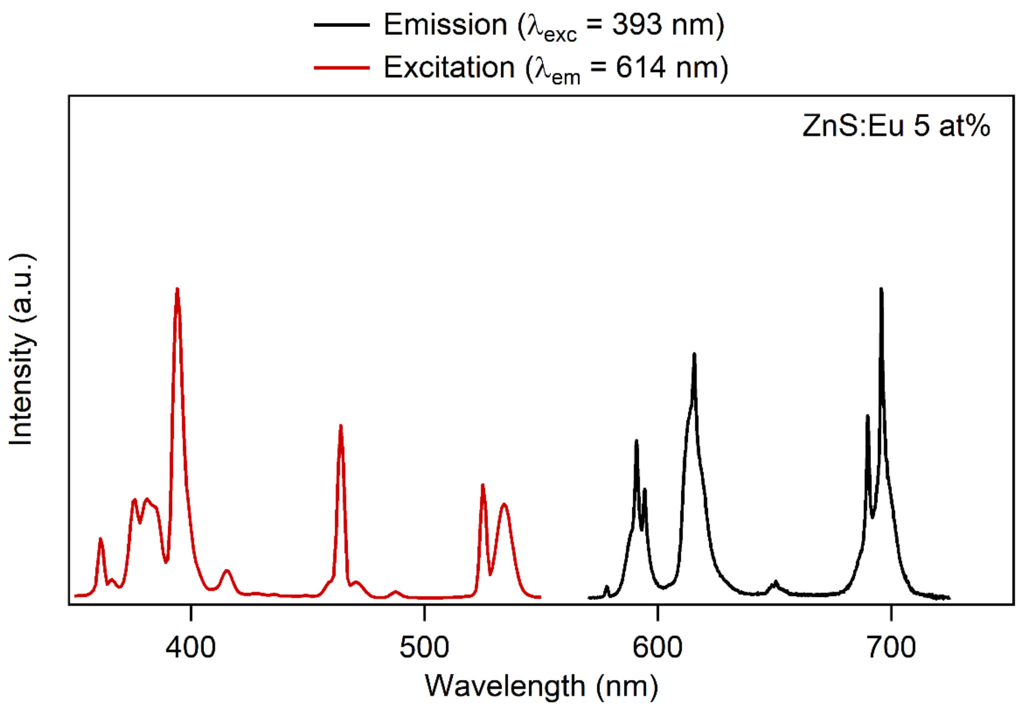

Figure S31. Emission spectrum (black line, λ_{exc}=393 nm) and excitation spectrum (red line, λ_{em}=614 nm) of and ZnS:Eu 5 at%.
